# Supplementary material for: Prevalence of educational needs and factors associated with educational severity among Rohingya refugee children in Cox’s Bazar, Bangladesh: A representative cross-sectional household survey
Source: PLOS Glob Public Health. 2026 Aug 31;6(8):e0007223. doi: 10.1371/journal.pgph.0007223 (PMC13528982; doi:10.1371/journal.pgph.0007223)
Supplement: Supplementary file 1 [file pgph.0007223.s003.pdf]

PLOS Global Public Health

Prevalence of educational needs and factors associated with educational severity among Rohingya refugee children in Cox's Bazar, Bangladesh: a representative cross-sectional household survey

--Manuscript Draft--

**Manuscript Number:**	PGPH-D-26-00678R1
**Article Type:**	Research Article
**Full Title:**	Prevalence of educational needs and factors associated with educational severity among Rohingya refugee children in Cox's Bazar, Bangladesh: a representative cross-sectional household survey
**Short Title:**	Factors associated with educational severity among Rohingya refugee children in Bangladesh
**Corresponding Author:**	Harry J Wilson, (B.BMed.Sci, MPH - Epidemiology, MGH, MIDI)<br>Monash University Faculty of Medicine Nursing and Health Sciences<br>Melbourne, Victoria AUSTRALIA
**Order of Authors:**	Harry J Wilson, (B.BMed.Sci, MPH - Epidemiology, MGH, MIDI)
	Nyanhial Yang, (B.BMed.Sci, MPH)
	Md Nazmul Karim, (MBBS, MPH, M Clin Epi, PhD, SFHEA)
**Keywords:**	Rohingya; Humanitarian Emergency; refugee; Refugee Camps; human rights
**Abstract:**	Background<br>Approximately 1.2 million Rohingya refugees currently reside among the 33 refugee camps in Cox's Bazar - Bangladesh, where the right to education remains a critical humanitarian concern. This study aimed to estimate the prevalence of Rohingya refugee children in educational need, elucidate predominant reasons among children in need, and identify factors associated with educational severity to inform targeted humanitarian relief efforts.<br>Methods<br>This study utilized participant data collected during the 2023 Joint Multi-Sectoral Needs Assessment - a representative cross-sectional household survey. Education-age children (3-18) were assigned a severity score categorized as stressed, severe, or extreme based on proxy responses capturing educational access, learning conditions, and protection factors. Ordinal logistic regression examined factors associated with increasing educational severity scores.<br>Results<br>Among 8,408 education age children surveyed from 2,959 households, 29.6% were in severe educational need and 10.1% were in extreme educational need. After adjustment, increasing educational severity scores were significantly associated with head of household age 45-59 (aOR= 1.29 [95%CI: 1.09–1.54]), head of household age 60+ (aOR= 1.31 [95%CI: 1.07–1.60]), female headed households (aOR= 1.33 [95%CI: 1.15–1.54]), early education age boys (aOR= 3.95 [95%CI: 3.21–4.85]) and teenage boys (aOR= 4.42 [95%CI: 3.73–5.24]) relative to primary school age boys, early education age girls (aOR= 4.23 [95%CI: 3.36–5.31]) and teenage girls (aOR= 19.06 [95%CI: 15.70–23.15]) relative to primary school age girls, primary school age girls (aOR= 0.82 [95%CI: 0.68–0.99]) relative to primary school age boys, and teenage girls (aOR= 3.54 [95%CI: 3.04–4.12]) relative to teenage boys.<br>Conclusion<br>Educational needs remain highly prevalent among Rohingya refugee children – particularly teenage girls. Local humanitarian authorities are currently providing targeted relief to those in educational need but these interventions are threatened by sweeping international funding cuts. Future longitudinal studies are required to monitor educational needs over time and further clarify causal pathways.
**Additional Information:**	
**Question**	**Response**
Financial Disclosure	The author(s) received no specific funding for this work.

Powered by Editorial Manager® and ProduXion Manager® from Aries Systems Corporation

Enter a financial disclosure statement that describes the sources of funding for the work included in this submission and the role the funder(s) played. This includes grants and any commercial funding of the work or authors.<br><br>This statement will be typeset if the manuscript is accepted for publication.<br><br>*Please review the submission guidelines and the instructions link below for detailed requirements and guidance.*	
**Competing Interests**<br><br>On behalf of all authors, disclose any competing interests that could be perceived to bias this work.<br><br>This statement will be typeset if the manuscript is accepted for publication.<br><br>*Please review the instructions link below and PLOS Global Public Health's competing interests policy to determine what information must be disclosed at submission.*	The authors have declared that no competing interests exist.
**Data Availability**<br><br>Before publication, Authors are required to make fully available and without restriction all data underlying their findings. Sharing the data underlying your findings increases the visibility, credibility, and impact of your work, while supporting transparency and future scientific discovery. Please see our PLOS Data Policy page for detailed information on this policy.	The de-identified participant-level dataset underlying the study results may be available upon reasonable request to UNHCR at https://microdata.unhcr.org/index.php/catalog/1128 subject to approval based on their data sharing policies, ethical requirements, and data use agreements.

A **Data Availability Statement**, detailing where the data can be accessed, is required at first submission. Insert your Data Availability Statement in the box below.<br><br>Please see the data reporting section of our submission guidelines for instructions on what you need to include in your Data Availability Statement. Examples of acceptable Data Availability Statements are included here for reference:<br>1. The minimal data set [and accompanying code, where generated] is available at [repository name] via [DOI hyperlink]<br>2. No data was generated by this study. The following existing data sources were used: [dataset name] from [dataset location] available via [web address or DOI hyperlink].<br>3. For Study Protocols: No datasets were generated or analysed during the current study. All relevant data from this study will be made available upon study completion.<br><br>*PLOS allows rare exemptions to address legal and ethical concerns. If you have legal or ethical restrictions, please use the box below to detail these in full sentences for the Journal team to consider.*<br><br>This statement will be typeset if the manuscript is accepted for publication.	

Powered by Editorial Manager® and ProduXion Manager® from Aries Systems Corporation

Manuscript Click here to access/download;Manuscript;Manuscript.docx

Prevalence of educational needs and factors associated with educational severity among Rohingya refugee children in Cox's Bazar, Bangladesh: a representative cross-sectional household survey

Harry J Wilson[1*], Nyanhial Yang[2], Md Nazmul Karim[1]
[1] School of Public Health and Preventive Medicine, Monash University, Melbourne, Victoria, Australia
[2] Murdoch Children's Research Institute, The Royal Children's Hospital, Melbourne, Victoria, Australia
[*] Corresponding author
Harry.J.Wilson@UNSWalumni.com

Abstract

Background

Approximately 1.2 million Rohingya refugees currently reside among the 33 refugee camps in Cox's Bazar - Bangladesh, where the right to education remains a critical humanitarian concern. This study aimed to estimate the prevalence of Rohingya refugee children in educational need, elucidate predominant reasons among children in need, and identify factors associated with educational severity to inform targeted humanitarian relief efforts.

Methods

This study utilized participant data collected during the 2023 Joint Multi-Sectoral Needs Assessment - a representative cross-sectional household survey. Education-age children (3-18) were assigned a severity score categorized as stressed, severe, or extreme based on proxy responses capturing educational access, learning conditions, and protection factors. Ordinal logistic regression examined factors associated with increasing educational severity scores.

Results

Among 8,408 education age children surveyed from 2,959 households, 29.6% were in severe educational need and 10.1% were in extreme educational need. After adjustment, increasing educational severity scores were significantly associated with head of household age 45-59 (aOR= 1.29 [95%CI: 1.09–1.54]), head of household age 60+ (aOR= 1.31 [95%CI: 1.07–1.60]), female headed households (aOR= 1.33 [95%CI: 1.15–1.54]), early education age boys (aOR= 3.95 [95%CI: 3.21–4.85]) and teenage boys (aOR= 4.42 [95%CI: 3.73–5.24]) relative to primary school age boys, early education age girls (aOR= 4.23 [95%CI: 3.36–5.31]) and teenage girls (aOR= 19.06 [95%CI: 15.70–23.15]) relative to primary school age girls, primary school age girls (aOR= 0.82 [95%CI: 0.68–0.99]) relative to primary school age boys, and teenage girls (aOR= 3.54 [95%CI: 3.04–4.12]) relative to teenage boys.

Conclusion

Educational needs remain highly prevalent among Rohingya refugee children – particularly teenage girls. Local humanitarian authorities are currently providing targeted relief to those in educational need but these interventions are threatened by sweeping international funding cuts. Future longitudinal studies are required to monitor educational needs over time and further clarify causal pathways.

Keywords: Rohingya; Humanitarian Emergency; Refugee; Refugee Camps; Human Rights;

Introduction

Eight years on from the mass displacement events in late 2017, approximately 1.2 million Rohingya refugees are currently sheltering within 33 congested camps of Cox's Bazar near the Bangladesh-Myanmar border [1].. The protracted nature of the Rohingya refugee crisis underscores the necessary operational transition from an acute emergency response to an enduring humanitarian program. Meeting the humanitarian needs of Rohingya refugees remains imperative for the international community but has met a critical juncture characterised by entrenched vulnerabilities, aid dependencies, concurrent domestic needs to support host communities, deteriorating humanitarian conditions within the camps, and a resurgence in targeted violence across the broader in Myanmar that has displaced an additional 150,000 Rohingya [1-7]. Although the contemporary humanitarian scenario has worsened, the international attention to the protracted crisis has waned [1,2,4,6,7]. The timing of these operational challenges precipitate amid a backdrop of changing geopolitical landscapes and receding humanitarian budgets that continues to threaten the delivery of essential humanitarian relief services for Rohingya refugees [1,3,5,8-10].

Approximately half of all Rohingya refugees sheltering among the camps in Cox's Bazar are education age children [1].. Consequentially, the demographic profile positions the education sector as a key cornerstone of the contemporary humanitarian response [1,5,10]. Beyond the direct individual benefits, ensuring the right to education is of paramount importance to protect vulnerable children, foster community resilience, build capacity for self-determination and strengthen prospective recovery efforts when circumstances allow safe voluntary repatriation [5,6,11-13]. A growing body of evidence highlights the pragmatic need for standardized education in emergency programs to incorporate nuanced age-gender responsive interventions that are co-designed with refugee communities, contextually framed within the lived experience of education age children they serve, and commensurate with the specific barriers they endure [14-19]. Additionally, the transition from childhood to adolescence may intensify gendered effects among school-age boys and girls that may differently interact with standardized education services [13,19,20]. This developmental period is particularly crucial for teenage girls, significantly influencing their physical, psychosocial, and emotional well-being that persist into adulthood [12,18]. Reciprocally, the implications of compromised educational attainment are compounded in protracted displacement settings such as the Rohingya refugee crisis where cumulative exposure over time threatens to not only widen but entrench gendered inequities [11,13,18,21].

Concerningly, prior analysis has identified the education sector as the leading reason for extreme humanitarian needs among Rohingya refugee households surveyed during the 2023 Joint Multi-Sectoral Needs Assessment (J-MSNA) [22]. Preliminary investigation foreshadowed gendered disparities and raised serious child protection concerns [22]. However, this analysis was restricted to extreme

educational needs only and did not disaggregate education sector severity scores by age, gender or underlying reasons that limits its ability to strategically inform sectoral programming [22].
Appling a more sensitive sector-specific investigation of the education sector using the same 2023 J-MSNA dataset, this study aimed to estimate the prevalence of Rohingya refugee children in educational need, evaluate the distribution of education sector severity by age, gender and attributable reason to intelligently inform specific interventions, and identify household vulnerability factors and demographic characteristics associated with educational severity scores to strengthen targeted relief efforts by local humanitarian authorities [23].

Methods

Study design, population and sampling strategy

This study draws upon anonymized participant data from the 2023 J-MSNA, a representative cross-sectional survey of Rohingya households within the 33 refugee camps of Cox's Bazar, used for secondary analysis [23]. The primary data collection was conducted by the REACH Initiative in conjunction with sector-specific authorities and local enumerators. The dataset was accessed on the 27th of March 2025 under license approved by UNHCR. The study population included all Rohingya refugee households registered within the 33 camps of Cox's Bazar [24,25]. An administratively complete sampling frame of registered households was compiled by camp authorities [24,25]. A total of 3,465 households were selected for data collection using stratified random sampling with camps as strata [24,25]. The number of households selected for sampling at each camp were guided by a single proportion sample size calculation that derived the minimum number of households needed to ensure camp-specific results could be concluded with a minimum level of precision (10% margin of error) at a 95% confidence level [24,25]. The sample size was adjusted for the finite household population that were eligible for selection at each camp and inflated by an additional 10% to mitigate non-response [24,25]. Although the study was primarily powered to enable camp specific results at the household-level, the achieved sample size provided sufficient precision for individual-level subgroup analyses by age and gender. Of the 3,465 households selected for sampling, a total sample size of 3,400 households were surveyed between August 27th and September 17th 2023 with individual data pertaining to 18,172 household members. Missingness was minimal (<1%) and these records were excluded from analysis.

Data collection

Data were collected through in-person household interviews administered by trained enumerators using a validated structured questionnaire that captured standardized humanitarian indicators and sector-specific items [24,25]. Gender-balanced field teams (comprising male and female interviewers) conducted interviews with an adult (age 18 or older) respondent of the same gender (self-identified)

[24,25]. Education status and reported reasons for educational need were provided by the adult respondent on behalf of each child within the household (proxy respondent), rather than by the children themselves. Responses were digitally recorded using the KoBo Collect application on mobile devices and routinely uploaded to a secure UNHCR server [24,25]. Raw participant data was cleaned according to the REACH Initiative's minimum standards protocol with a final de-identified dataset provided for statistical analysis [24,25]. Verbal informed consent was secured from all household respondents and formally recorded in the deidentified dataset. Respondents were informed of the survey's objectives, the voluntary nature of their participation, measures to protect response confidentiality and prerogative to decline any question or discontinue the interview at any time. Enumerators were educated on the principles of participant safeguarding and trained to provide formal referral protocols upon discovery of urgent protection or health concerns.

Variables

The primary outcome was education severity score assigned to each education age child (age 3-18) based on proxy responses to questionnaire items provided by an adult respondent on behalf of each education age child within the household that captured the four dimensions of educational access, learning conditions, protection and aggravating circumstances. Education severity scores were assigned based on recommended criteria outlined in the Global Education Cluster (UNICEF and Save the Children) severity classification manual, Joint Intersectoral Analysis Framework (JIAF) and Multi-Sectoral Needs Index (MSNI) [24,26,27]. Severity was scored according to the JIAF five-point intersectoral severity classification scale ranging from minimal (score=1) to catastrophic (score=5). The five-point intersectoral severity scale was operationalised as a three-point scale in line with J-MSNA methods due to contextual factors and sampling units rather than inferential constraints that may otherwise compromise the construct validity of the established classification scheme.

The minimal severity category (score=1) could not be assigned due to the absence of formal education services within the camps. Hence, the lowest possible score that could be assigned reflected regular attendance at a non-formal learning centre corresponding to a severity score of stressed (score=2) rather than minimal (score=1). Conversely, the catastrophic severity score (score=5) requires aggregate response-level data indicating complete sectoral collapse and systemic exclusion rather than individual learning conditions and reasons for not accessing education as reported by proxy respondents on behalf of the household.

The operationalized three-point scale categorised each education age child as either stressed (score=2), severe (score=3) or extreme (score=4). The restricted three-point scale retains the same ordinal severity distinctions of the established five-point intersectoral severity scale, supporting meaningful comparisons of educational severity scores across subgroups. The lowest possible score of stressed

(score=2) was defined as accessing non-formal education in a protected environment offering acceptable learning conditions.

Severe (score=3) was classified as either not accessing education due to non-aggravating circumstances, or accessing non-formal education in a protected environment but experiencing inadequate learning conditions or minor safety issues that do not present an immediate threat to the child's wellbeing. Inadequate learning conditions included curriculum is not adapted, displacement induced change, lack of qualified teachers, lack of teaching/learning material, language barriers, overcrowding, and poor water/sanitation/hygiene conditions. Minor safety issues included physical or verbal bullying between students, physical punishment from teachers, teaching staff unqualified in psychosocial support, and unsafe infrastructure

Extreme (score=4) was defined as either not accessing education services due to aggravating circumstances, or attending an unprotected learning environment due to serious safety issues that endanger the child's well-being. Aggravating circumstances correspond to the most severe accessibility barriers such as marriage or pregnancy, child paid and unpaid labour to support the household instead of attending school, recruitment by armed groups, discrimination, and safety threats whilst travelling to or attending school [24,27]. Unprotected learning environment included armed group recruitment, school attacks, gender based or sexual violence, and discrimination based on the child's ethnic, gender or religious identity [26,27]. The underlying reason for each child's education severity score were utilised as secondary outcomes to evaluate predominate underlying reasons for educational severity scores and their distribution across age-gender subgroups.

Individual characteristics of education age children and household vulnerability factors evaluated for association with educational needs were identified from previous J-MSNA research, Refugee Influx Emergency Vulnerability Assessments (REVA), and funding impact analysis conducted by the World Food Programme [8,9,28,29]. Age groups were assigned according to previous camp levels findings and appropriate cutoff points identified from trends observed over increasing age [30]. Subgroups among education age children (age 3-18) included early education age children (age 3-4), primary school age children (age 5-11) and teenage children (age 12-18). The rationale for the selected age cutoff point used to distinguish primary school age children (age 5-11) from teenage children (age 12-18) was based on mitigating contamination of age-groups from effect heterogeneity observed during linear trend analysis. Head of household age was categorized into 15-year age groups. Marital status was collapsed into a binary variable that included married and single participants (no partner/separated/divorced/widowed) due to limited observations. Head of household disability status was classified as a binary variable corresponding to participants citing either "a lot of difficulty" or "cannot do it at all" to any of the six functional domains as per the Washington Group Short Set (WG-SS) guidelines [31]. Monthly household income per capita (household members) was categorized according to quartiles and utilized to investigate associations with financial vulnerability.

Statistical analysis

All results were computed using Stata version 19.5 survey functions that accounted for the stratified sampling design, adjusted for the clustering of education age children within the same household and controlled for differential probability of household selection between the camps (strata) using survey weights computed as the inverse probability of selection [32].

Descriptive statistics were used to analyse demographic characteristics of sampled households and education age children. Weighted frequencies and percentages were reported for categorical variables. Median and interquartile range (IQR) or mean and standard deviation (SD) were reported for continuous variables depending on symmetry. Prevalence estimates of children and households experiencing educational needs were reported as weighted percentages with 95% confidence intervals (95%CIs) for precision.

Education severity scores and their attributable reasons were disaggregated by age and gender. The distribution of severity scores and reported reasons were assessed over increasing age and by gender to assess linear trends. Ordinal logistic regression analysis was conducted to investigate factors associated with increasing education sector severity scores and the assumption of proportional odds was tested. Unadjusted odds ratios (OR) and adjusted odds ratios (aOR) were reported for bivariate and multivariate estimates respectively. A threshold value of $p \leq 0.05$ was used to conclude statistical significance and 95%CIs were reported for precision using design-adjusted standard errors computed via Taylor series linearization.

Potential effect modification was considered between head of household gender and marital status to investigate concentrated effects among single female headed households. Two-way age and gender interactions among education age children (age-gender and gender-age) were investigated using both stratified analysis and interaction terms. Gender-age interactions were investigated using stratified (by gender) analysis reporting the relative risk associated with age groups among boys and among girls. Age-gender interactions were investigated using interaction terms to evaluate the relative risk associated with girls relative to boys within the same age group. Pairwise correlation and variance inflation factor (VIF) analysis were conducted to evaluate potential problematic multicollinearity.

Results

Participant characteristics

Among the 3,400 total households sampled, 441 households did not contain education age children (age 3-18) and were excluded from subsequent analysis (Table 1). A total of 8,408 education age children were surveyed from the remaining 2,959 households across the 33 camps. The number of households without education age children (n=441) did not significantly differ between the camps ($p > 0.05$). The

proportional contribution of each camp to the overall sample showed minimal variation (mean change= +0.5%; range: -12.6% to +8.7%) before (N=3,400) and after (N=2,959) the exclusion of households without education age children. The number of households and education age children sampled per camp was approximately normally distributed with a mean of 90 households (SD= 35; Table 1) and 255 education age children (SD= 95; Table 1) respectively. The median head of household age was 39 years (IQR: 30 – 50; Table 1) and the majority of households were male headed (79.8%; Table 1). Approximately half of surveyed children were aged between 5-11 years (47.4%; Table 1) and the proportion of boys sampled (50.3%; Table 1) was slightly higher than the proportion of girls (49.7%; Table 1). Less than two-thirds of education age children were enrolled and regularly attending informal education within the camps (60.3%; Table 1). The prevalence of children in educational need was 39.7% (95% CI: 38.4% – 41.0%) and almost two-thirds of households contained at least one child in educational need (65.7% [95% CI: 63.8% – 67.5%]).

Table 1. Characteristics of sampled households (N= 2,959) and education age children (N= 8,408)

	**Summary***
**Households per Camp, Mean (N= 2,959)**	90 (SD: 35)
**Education Age Children per Camp, Mean (N= 8,408)**	255 (SD: 95)
**Head of Household Age, Median (N= 2,959)**	39 [IQR: 30 – 50]
18-29	688 (23.2%)
30-49	1414 (47.8%)
50+	857 (29.0%)
**Head of Household Gender (N= 2,959)**	
Male	2361 (79.8%)
Female	598 (20.2%)
**Education Age Children per Household, Median (N= 2,959)**	3 [IQR: 2 – 4]
1-2	1465 (49.5%)
3-5	1284 (43.4%)
6-9	210 (7.1%)
**Age, median (N= 8,408)**	10 [IQR: 6 – 14]
3-4	1,165 (13.9%)
5-11	3,985 (47.4%)
12-18	3,258 (38.7%)
**Gender (N= 8,408)**	
Boys	4233 (50.3%)
Girls	4175 (49.7%)
**Education Sector Severity (N= 8,408)**	
Stressed (Severity Score=2)	5070 (60.3%)
Severe (Severity Score=3)	2488 (29.6%)
Extreme (Severity Score=4)	850 (10.1%)
**Prevalence of Educational Need[a]**	
Children in Educational Need (N= 8,408)	39.7% [95% CI: 38.4% – 41.0%]
Households in Educational Need (N= 2,959)	65.7% [95% CI: 63.8% – 67.5%]

* Estimates are relative to either the total number of households with education age children (N= 2,959) or the total number of education age children (N= 8,408). Summary statistics are weighted frequency and weighted percentages for categorical variables, weighted median (IQR) for asymmetrically distributed continuous variables, weighted mean (SD) for symmetrical continuous variables, or weighted population prevalence (%) and 95% CIs for outcome variables.

[a] Children in educational need was classified as severe or extreme education severity scores (severity score=3+) as per MSNI methods [24]. Children in education need defined as per JIAF and education cluster guidelines - Education age children in the areas affected by crisis who do not have access to protective education and acceptable learning conditions, which can negatively impact (i) their physical and psychosocial wellbeing, (ii) cognitive development, and (iii) their ability to meet their future needs [26,27]. Households in educational need categorised as those with at least one child in educational need as per MSNI methods [24].

Educational Severity by age and gender

The distribution of education sector severity scores (Fig 1A) and their attributable reasons (Fig 1B) disaggregated by age-group and gender demonstrated three clear patterns: (i) substantial unmet educational need in early childhood driven by enrolment barriers, (ii) high attendance among children aged 5–11 with limited extreme need, and (iii) a sharp decline in enrolment after age 12 with pronounced gender disparities. Approximately half (54.3%; Fig 1A) of early education age children (3-4 years) were enrolled and regularly attending informal education with similar results observed among boys (52.4%) and girls (56.1%). A small proportion of early education age children were found to be experiencing extreme educational need mostly attributable to “protection risks while commuting to school” (4.3%; Fig 1B). Most primary school age children (5-11) were enrolled and regularly attending informal education (83.3%; Fig 1A) with similar results observed between boys (81.8%; Fig 1A) and

girls (84.9%; Fig 1A). Comparatively, only a third (34.4%; Fig 1A) of teenage children age 12-18 were enrolled and regularly attending informal education with significant heterogeneity observed between boys (49.1%; Fig 1A) and girls (20.1%; Fig 1A). Most teenage children were experiencing severe educational needs (44.9%; Fig 1A) with greater prevalence observed among teenage girls (50.6%; Fig 1A) compared to boys (38.9%; Fig 1A).

Fig 1A. Education severity scores among education age children by age group and gender.

Fig 1B. Attributable reason for educational needs among children (age 3-18) by age group and gender.

[a] Rarely reported reasons (<1%) for severe educational needs among all cohorts were recoded as "other" rather than individually categorised for visual clarity. "Other" includes the following reasons and percentages:
Children Age 3-4: Lack of Schools (0.3%), Language Issues (1.3%), Decline/Do not Know (0.6%)
Boys Age 3-4: Lack of Schools (0.2%), Language Issues (1.5%), Decline/Do not Know (0.7%)
Girls Age 3-4: Lack of Schools (0.4%), Language Issues (1.2%), Decline/Do not Know (0.4%)
Children Age 5-11: Illness (0.1%), Lack of Schools (0.2%), Decline/Do not Know (0.5%)
Boys Age 5-11: Cannot Afford Education Costs (0.1%), Illness (0.1%), Lack of Schools (0.1%), Decline/Do not Know (0.5%)
Girls Age 5-11: Illness (0.1%), Lack of Schools (0.4%), Decline/Do not Know (0.4%)
Children Age 12-18: Cannot Afford Education Costs (0.2%), Child Uninterested (0.3%), Home Based Learning (0.4%), Inadequate Curriculum (0.2%), Decline/Do not Know (2.4%)
Boys Age 12-18: Cannot Afford Education Costs (0.3%), Child Uninterested (0.5%), Home Based Learning (0.7%), Inadequate Curriculum (0.1%), Decline/Do not Know (1.8%)
Girls Age 12-18: Cannot Afford Education Costs (0.1%), Child Uninterested (0.1%), Home Based Learning (0.1%), Illness (0.1%), Inadequate Curriculum (0.2%), Decline/Do not Know (3.0%)

The analysis of education severity scores over increasing age and by gender illustrated similar severity profiles among boys and girls between ages 3-11, followed by a sharp decline in the proportion of children enrolled and regularly attending school among teenagers (ages 12-18) with substantial gender disparities (Fig 2A). A rapid rise in severe educational needs was observed among adolescent girls age 12-15 (Fig 2A), predominantly attributable to “education is not a priority” and “inadequate male-female separation” (Fig 2B). A linear trend between extreme educational needs and increasing age was evident among teenage girls age 11-18, mainly attributable to “child at home supporting the household instead”, and not attending due to “protection risks at or commuting to school” during ages 12-15, followed by an exponential rise in “marriage or pregnancy” among teenage girls age 16-18 (Fig 2B). Educational needs among teenage boys remained relatively constant until the ages of 16-18 predominantly attributable to “education is not a priority” (Fig 2B) and “child at home supporting the household instead” (Fig 2B).

Fig 2A. Education severity scores over increasing age (3-18) and by gender (children, boys, girls).

Fig 2B. Reported reason for educational needs over increasing age (3-18) and by gender (children, boys, girls).

Household vulnerability factors and individual characteristics associated with educational severity

In unadjusted analysis, increasing educational severity scores were associated with older head of households, female headed households, head of households living with a disability, age-groups among boys, age-groups among girls, and girls relative to boys within the same age group.

After adjustment in multivariable analysis, increasing educational severity scores were significantly associated with head of household age 45-59 (aOR= 1.29 [95%CI: 1.09–1.54]), head of household age 60+ (aOR= 1.31 [95%CI: 1.07–1.60]), female headed households (aOR= 1.33 [95%CI: 1.15–1.54]), early education age boys (aOR= 3.95 [95%CI: 3.21–4.85]) and teenage boys (aOR= 4.42 [95%CI: 3.73–5.24]) relative to primary school age boys, early education age girls (aOR= 4.23 [95%CI: 3.36–5.31]) and teenage girls (aOR= 19.06 [95%CI: 15.70–23.15]) relative to primary school age girls, primary school age girls (aOR= 0.82 [95%CI: 0.68–0.99]) relative to primary school age boys, and teenage girls (aOR= 3.54 [95%CI: 3.04–4.12]) relative to teenage boys

The multivariate model indicated adequate fit over a null model ($p<0.001$) and did not violate the assumption of proportional odds. No evidence of problematic multicollinearity was observed, all pairwise correlations between independent variables were below 0.7 and the mean VIF was 1.5 with no extreme outliers ($VIF_{max}<10$). No statistically significant evidence of an interaction on the multiplicative scale was found between female headed households and marital status ($p >0.05$) with main effects reported in consequence.

Table 2. Individual factors and household characteristics associated with higher educational severity scores.

	Summary	Odds Associated with Higher Educational Severity Scores			
	(N=8,408)	Unadjusted		Adjusted	
	n (%)	OR (95%CI)	p-value	aOR (95%CI)	p-value
**Head of Household Age**					
18-34	2,521 (30.0%)	1.00	–	1.00	–
35-44	2,577 (30.6%)	**1.28 (1.10–1.47)**	**<0.001***	0.92 (0.77–1.09)	0.319
45-59	2,225 (26.5%)	**2.39 (2.08–2.75)**	**<0.001***	**1.29 (1.09–1.54)**	**0.003***
60+	1,085 (12.9%)	**2.80 (2.36–3.31)**	**<0.001***	**1.31 (1.07–1.60)**	**0.008***
**Head of Household Gender**					
Male	6,925 (82.4%)	1.00	–	1.00	–
Female	1,483 (17.6%)	**1.50 (1.31–1.72)**	**<0.001***	**1.33 (1.15–1.54)**	**<0.001***
Female: Married[a]	780 (9.3%)	1.00	–	1.00	–
Female: Single/Divorced/Widowed[a]	703 (8.3%)	1.15 (0.91–1.46)	0.241	1.01 (0.78–1.30)	0.960
**Head of Household Disability Status**					
No Disability	6,972 (82.9%)	1.00	–	1.00	–
Living with a Disability	1,436 (17.1%)	**1.19 (1.03–1.37)**	**0.017***	1.03 (0.88–1.21)	0.686
**Monthly Household Income (per capita)[b]**					
1st Quartile (<$US 12.5)	2,385 (28.4%)	1.00	–	1.00	–
2nd Quartile ($US 12.5–17.49)	2,379 (28.3%)	0.99 (0.86–1.14)	0.903	1.09 (0.93–1.28)	0.280
3rd Quartile ($US 17.5–24.99)	2,174 (25.9%)	0.90 (0.77–1.04)	0.148	0.95 (0.81–1.12)	0.543
4th Quartile (≥$US 25)	1,470 (17.4%)	1.08 (0.92–1.26)	0.363	1.13 (0.95–1.35)	0.157
**Boys: Age Group**					
Boys: Age 5-11	2,053 (24.4%)	1.00	–	1.00	–
Boys: Age 3-4	579 (6.9%)	**3.84 (3.14–4.70)**	**<0.001***	**3.95 (3.21–4.85)**	**<0.001***
Boys: Age 12-18	1,601 (19%)	**4.82 (4.09–5.68)**	**<0.001***	**4.42 (3.73–5.24)**	**<0.001***
**Girls: Age Group**					
Girls: Age 5-11	1,933 (23%)	1.00	–	1.00	–
Girls: Age: 3-4	585 (7%)	**4.12 (3.30–5.14)**	**<0.001***	**4.23 (3.36–5.31)**	**<0.001***
Girls: Age 12-18	1,657 (19.7%)	**21.23 (17.56–25.67)**	**<0.001***	**19.06 (15.70–23.15)**	**<0.001***
**Age 3-4: Gender**					
Age 3-4: Boys	579 (6.9%)	1.00	–	1.00	–
Age 3-4: Girls	585 (7%)	0.87 (0.70–1.09)	0.221	0.88 (0.70–1.10)	0.263
**Age 5-11: Gender**					
Age 5-11: Boys	2,053 (24.4%)	1.00	–	1.00	–
Age 5-11: Girls	1,933 (23%)	**0.81 (0.67–0.98)**	**0.033***	**0.82 (0.68–0.99)**	**0.044***
**Age 12-18: Gender**					
Age 12-18: Boys	1,601 (19%)	1.00	–	1.00	–
Age 12-18: Girls	1,657 (19.7%)	**3.58 (3.07–4.17)**	**<0.001***	**3.54 (3.04–4.12)**	**<0.001***

* Statistically significant at $p \leq 0.05$.

[a] Additional estimates from a duplicate model with interaction terms applied to investigate effect modification among single/divorced/widowed female headed households relative to married female headed households. Note that estimates reported for female headed households represent the main effects among all female headed households rather than the subgroup of married female headed households. All multivariable estimates were adjusted for main effects only.

[b] Monetary value in 2023 US dollars.

Discussion

The demographic characteristics of households with education age children closely mirror those of all sampled households that prior analysis demonstrated to be strongly aligned with UNHCR's database of all Rohingya refugees registered within the camps [22]. The alignment observed between the sample and population characteristics strongly support the representativeness of participants and hence generalisability of our results [22].

Despite ongoing humanitarian relief efforts by local authorities, the findings highlight that almost two-thirds of households, and 2-in-every-5 children age 3-18 were in educational need. Disaggregation by age group illustrated the higher prevalence of educational needs among early education age children (age 3-4) and teenage children (age 12-18). Further disaggregation by gender evidenced the similarities among boys and girls during childhood (age 3-11), followed by the rapid divergence of educational needs during adolescence (age 12-18). These age-gender dynamics are best illustrated by the distribution of severity scores and their attributable reasons over increasing age that further evidenced the gendered similarities during childhood and contrasting linear trends observed during adolescence that may reflect the emergence of gendered interactions that differentiate teenage boys and teenage girls. These descriptive interpretations are supported by the logistic regression results that revealed a sharp increase in the odds associated with teenage girls relative to both primary school age girls and teenage boys.

The associations observed between educational needs and individual age-gender characteristics persisted with limited attenuation after adjustment for common household vulnerability factors. The comparative strength of association with individual-level severity scores among education age children may suggest the broader influence of exogenous structural factors and systemic challenges within the education sector such as restrictive enrolment policies experienced by teenage children that may provoke the increasing trend in educational severity scores observed from age 11 onwards. This interpretation regarding exogenous structural constraints are consistent with findings from previous sector-specific analysis that particularly highlight the limited learning opportunities for teenage girls [5,14,15,21,28]. Similarly, our findings align with education-in-emergency frameworks that conceptualise schooling as both an important humanitarian service and protective system, with an emphasis on safeguarding the heightened vulnerability among adolescent children. In contrast, the elevated odds associated with female headed households, young adult (18-24) headed households and elderly (age 45+) headed households may suggest the influence of survival pressures that could potentially provoke households to deprioritise their children’s education. These interpretations are supported by the distribution of reported reasons for educational need, which are highly concentrated among teenage girls. The trends regarding deprioritization and deteriorating severity scores highlight the plausible intersections between the education sector and the broader survival pressures within the camps that may position households to adopt negative livelihood coping strategies [33,34]. In addition,

the high proportion of teenage girls in extreme educational need reportedly due to childhood marriage and early pregnancy emphasize the close links between deteriorating educational needs and serious child protection issues that require urgent remediation efforts.

Limitations

The findings should be interpreted within the context of multiple strengthens and limitations. The stratified random sampling, survey weighting and face-to-face interviews enhance representativeness of participants. However, the findings are predominantly limited by the use of self-reported data collected from proxy respondents on behalf of the household that may consequentially introduce measurement bias and undermine the accuracy of the results.

The cross-sectional design limits causal inference and observed associations may reflect unmeasured confounding such as complex developmental issues that may modulate individual abilities to engage with the education sector. Additionally, several key determinants of educational access—such as learning-centre proximity, teacher availability, household mobility restrictions, and camp-level security conditions were not directly measured.

Finally, sensitive protection-related barriers (e.g., marriage, pregnancy, exploitation, safety threats) may be under-reported due to stigma and proxy reporting, potentially leading to conservative estimates of extreme need and potentially lower odds ratios among girls that are more likely to experience both sensitivities and stigmatisation.

Implications for practice

Our findings present numerous implications for humanitarian programming within the education sector. The age- and gender-specific gradients identified in this study suggest that education programming should be consolidated among early education and primary school age children, and differentiated among teenage boys and teenage girls to strategically target gendered protection issues, sociocultural barriers and accessibility constraints. In response, over 20 humanitarian projects coordinated by lead agencies, international authorities and non-government organisations are currently providing relief to Rohingya refugee children in educational need [5]. These projects include school feeding programs for disadvantaged students, reuptake programs for out-of-school children, accelerated learning programs for students who are at risk of falling further behind and restructuring of learning centres to provide female-only classrooms that redress sociocultural barriers among teenage girls [4,10]. The continuity of these projects are critical to affirm the right to education among Rohingya refugee children yet remain desperately underfunded at just 15% of their stated requirements [35]. The detrimental impacts of funding cuts among the shock-prone education sector are already evident, provoking the closure of 43% of learning facilities and denying access to education for approximately 200,000 Rohingya children [34,36]. Rapid restructuring efforts in conjunction with community adaptation have secured the gradual

recovery of the education sector [34]. However, these gains remain fragile and unable to deliver the sustained relief that the protracted crisis and their multidimensional complexities demand [10,34].

Conclusion

Educational needs remain highly prevalent among Rohingya refugee children in Cox's Bazar. Approximately a third of children age 3-18 were found to be in severe educational need and one in ten were experiencing extreme educational need. The findings reveal pronounced age-gender dynamics with teenage girls experiencing markedly higher educational needs, consistent with reported barriers related to deprioritisation, household survival pressures, sociocultural constraints and serious child protection concerns. Addressing the educational needs of Rohingya refugee children remains imperative to prevent gendered inequities, foster community resilience and strengthen recovery prospects from the protracted crisis. Ameliorating the scale of children in educational need, their complexities and intersectoral dependencies requires strategic, intensive and sustained humanitarian relief efforts. Future longitudinal and qualitative research is needed to redress study design constraints, closely monitor responses to educational needs over time, better capture sensitive protection-related barriers to education in consultation with community, and clarify causal pathways involving household vulnerability factors and individual age-gender characteristics that may act to influence educational severity scores.

Acknowledgements

The authors wish to formally acknowledge those whose collective efforts have made this research article possible. Specifically, the Inter-Sector Coordination Group (ISCG), UNHCR, IOM, ECHO, the REACH Initiative, enumerators, other study personnel, and household respondents who graciously donated their time.

References

1. Inter-Sector Coordination Group (ISCG). Recalibrating the Rohingya Response: 2025-26 Joint Response Plan - Rohingya Humanitarian Crisis, January 2026 - December 2026. 2026. Available from: https://reliefweb.int/report/bangladesh/recalibrating-rohingya-response-2025-26-joint-response-plan-rohingya-humanitarian-crisis.
2. United Nations Human Rights Council. Situation of human rights of Rohingya Muslims and other minorities in Myanmar. 2025. Available from: https://digitallibrary.un.org/record/4084836.
3. Médecins Sans Frontières. The illusion of choice: Rohingya voices echo from the camps. 2025. Available from: https://reliefweb.int/report/bangladesh/illusion-choice-rohingya-voices-echo-camps.
4. Inter-Sector Coordination Group (ISCG). Bangladesh: Rohingya Refugee Response - Flash Appeal for 150,000 New Arrivals (June - December 2025). 2025. Available from: https://reliefweb.int/report/bangladesh/bangladesh-rohingya-refugee-response-flash-appeal-150000-new-arrivals-june-december-2025.

5. Inter-Sector Coordination Group (ISCG). Bangladesh: Rohingya Humanitarian Crisis Joint Response Plan January 2025 - December 2026. 2025. Available from: https://reliefweb.int/report/bangladesh/bangladesh-rohingya-humanitarian-crisis-joint-response-plan-january-2025-december-2026.
6. United Nations General Assembly. Situation of human rights of Rohingya Muslims and other minorities in Myanmar: report of the United Nations High Commissioner for Human Rights. 2025. Available from: https://digitallibrary.un.org/record/4092194/files/A_HRC_60_20-EN.pdf.
7. Borak Z, Haque S. Rohingya refugees in Bangladesh should be allowed to work. Lancet Psychiatry. 2026;13(5). doi: 10.1016/s2215-0366(26)00055-6.
8. World Food Programme (WFP). Impact of Cuts - Bangladesh: Outcome of 2023 pilot study: Impact of funding shortfalls on beneficiaries. 2024. Available from: https://reliefweb.int/report/bangladesh/impact-cuts-bangladesh-outcome-2023-pilot-study-impact-funding-shortfalls-beneficiaries-august-2024.
9. The World Bank, World Food Programme (WFP). Impact of Food Ration Cuts on the Displaced Rohingya Population in Cox’s Bazar. 2024. Available from: https://reliefweb.int/report/bangladesh/bangladesh-impact-food-ration-cuts-displaced-rohingya-population-coxs-bazar-may-2024.
10. Inter-Sector Coordination Group (ISCG). Rohingya Refugee Response Urgent priorities: Addressing the Most Pressing Needs of the Joint Response Plan. 2025. Available from: https://reliefweb.int/report/bangladesh/bangladesh-rohingya-refugee-response-urgent-priorities-addressing-most-pressing-needs-joint-response-plan-june-december-2025.
11. World Health Organization. World report on promoting the health of refugees and migrants: monitoring progress on the WHO global action plan. 2026. Available from: https://iris.who.int/handle/10665/384927.
12. World Health Organization. WHO guideline on preventing early pregnancy and poor reproductive outcomes among adolescents in low- and middle-income countries. 2025. Available from: https://iris.who.int/handle/10665/381100.
13. Das J, Prima MM, Rimu FH, Hossain P, Das T, Nesa F, et al. Mental Health and Wellbeing of Rohingya Refugees: A Scoping Review. Journal of Racial and Ethnic Health Disparities. 2026. doi: 10.1007/s40615-026-02982-4.
14. REACH. Assessment of the Education Sector response to the Rohingya crisis - Rohingya refugee response. 2021. Available from: https://reliefweb.int/report/bangladesh/bangladesh-assessment-education-sector-response-rohingya-crisis-rohingya-refugee.
15. Cox's Bazar Education Sector. Joint Education Needs Assessment: Rohingya Refugee in Cox’s Bazar - June 2018. 2018. Available from: https://reliefweb.int/report/bangladesh/joint-education-needs-assessment-rohingya-refugee-cox-s-bazar-june-2018.
16. Education Cannot Wait. Holistic learning outcomes measurement handbook for education in emergencies and protracted crises. 2025. Available from: https://reliefweb.int/report/world/holistic-learning-outcomes-measurement-handbook-education-emergencies-and-protracted-crises.
17. Inter-agency Network for Education in Emergencies (INEE). INEE Minimum Standards for Education: Preparedness, Response, Recovery (2024 Edition). 2024. Available from: https://reliefweb.int/report/world/inee-minimum-standards-education-preparedness-response-recovery-2024-edition.
18. Inter-agency Network for Education in Emergencies. Mind the Gap 3: Equity and Inclusion in and through Girls' Education in Crisis. 2023. Available from: https://reliefweb.int/report/world/mind-gap-3-equity-and-inclusion-and-through-girls-education-crisis-enpt.
19. Hossain MA, Zablotska-Manos I. Family planning awareness and contraceptive use among young Rohingya refugee women: A representative cross-sectional study in Cox's Bazaar, Bangladesh. Womens Health (Lond). 2026;22. doi: 10.1177/17455057261416251.
20. Iiyama S, Izutsu T, Miyamoto Y, Yesmin S, Begum T, Mariam E, et al. Effectiveness of community-based mental health and psychosocial support through the Paracounsellor model for Rohingya refugees in Bangladesh. Public Health in Practice. 2026;11. doi: 10.1016/j.puhip.2026.100799.

21. Østby G, Gjerløw H, Karim S, Dunlop E. Left Further Behind after the COVID-19 School Closures: Survey Evidence on Rohingya Refugees and Host Communities in Bangladesh. Journal on Education in Emergencies. 2023;9(1). doi: 10.33682/a1zn-5nda.
22. Wilson HJ. Drivers and determinants of extreme humanitarian needs among Rohingya refugee households: Evidence from UNHCR's multi-sectoral needs analysis. PLOS ONE. 2025;20(12):e0331727. doi: 10.1371/journal.pone.0331727.
23. United Nations High Commissioner for Refugees (UNHCR), International Organization for Migration (IOM), Directorate-General for European Civil Protection and Humanitarian Aid Operations (ECHO). Bangladesh: Joint Multi Sector Needs Assessment: Cox's Bazar, Rohingya Refugee Response – 2023, Refugees. 2023. Available from: https://microdata.unhcr.org/index.php/catalog/1128.
24. REACH. Bangladesh J-MSNA - MSNI Methodological Overview. 2023 [cited 2025 Apr 4]. Available from: https://repository.impact-initiatives.org/document/impact/474ac9e5/BGD2301_2023-J-MSNA_-MSNI-Methodological-overview_Jan-2024.pdf.
25. REACH. Research Terms of Reference: Joint Multi-Sector Needs Assessment MSNA 2023, Bangladesh. 2023 [cited 2025 Apr 4]. Available from: https://reliefweb.int/report/bangladesh/research-terms-reference-joint-multi-sector-needs-assessment-msna-2023-bgd2301-bangladesh-august-2023-v-1.
26. United Nations Office for the Coordination of Humanitarian Affairs (UNOCHA). Joint and Intersectoral Analysis Framework (JIAF) 2 Technical Manual. 2024. Available from: https://reliefweb.int/report/world/joint-and-intersectoral-analysis-framework-jiaf-2-technical-manual-july-2024.
27. Global Education Cluster. Calculating Education Cluster People in Need (PiN). 2024. Available from: https://www.educationcluster.net/calculating-people-need.
28. Inter-Sector Coordination Group (ISCG). Joint Multi-Sector Needs Assessment (J-MSNA): Bangladesh Rohingya Refugees - December 2021. 2022. Available from: https://reliefweb.int/report/bangladesh/joint-multi-sector-needs-assessment-j-msna-bangladesh-rohingya-refugees-december-2021.
29. World Food Programme (WFP). Bangladesh: Refugee Influx Emergency Vulnerability Assessment (REVA-8) Report, June 2025. 2025. Available from: https://reliefweb.int/report/bangladesh/bangladesh-refugee-influx-emergency-vulnerability-assessment-reva-8-report-june-2025.
30. Inter-Sector Coordination Group (ISCG). Joint Multi-Sector Needs Assessments (J-MSNA) Factsheet - Refugee Camp-level findings - December. 2023. Available from: https://reliefweb.int/report/bangladesh/bangladesh-joint-multi-sector-needs-assessment-j-msna-camp-level-findings-december-2023.
31. The Washington Group on Disability Statistics. The Washington Group Short Set on Functioning (WG-SS). 2022 [cited 2025 Apr 4]. Available from: https://www.washingtongroup-disability.com/question-sets/wg-short-set-on-functioning-wg-ss/.
32. StataCorp. Survey Data Reference Manual. 2025. Available from: https://www.stata.com/bookstore/survey-data-reference-manual/.
33. World Food Programme (WFP). Livelihood Coping Strategies Indicator for Food Security: List of strategies and their definitions. 2023 [cited 2025 June 6]. Available from: https://docs.wfp.org/api/documents/WFP-0000147820/download/.
34. United Nations Office for the Coordination of Humanitarian Affairs (OCHA). Global Humanitarian Overview 2026: Rohingya Joint Response Plan (JRP). 2026 [cited 2026 Jan 8]. Available from: https://humanitarianaction.info/document/global-humanitarian-overview-2026/article/rohingya-joint-response-plan-jrp-1.
35. Inter-Sector Coordination Group (ISCG). Bangladesh: Rohingya Humanitarian Crisis Joint Response Plan 2025-26 funding update as of 31 October 2025. 2025. Available from: https://reliefweb.int/report/bangladesh/bangladesh-rohingya-humanitarian-crisis-joint-response-plan-2025-26-funding-update-31-october-2025.

36. United Nations Children's Fund (UNICEF). Humanitarian Action for Children 2026 - Bangladesh. 2026. Available from: http://reliefweb.int/report/bangladesh/humanitarian-action-children-2026-bangladesh.

Supporting information

S1 Document. STROBE checklist for cross-sectional studies.

Fig 1A

Click here to access/download;Figure;Fig 1A.tif

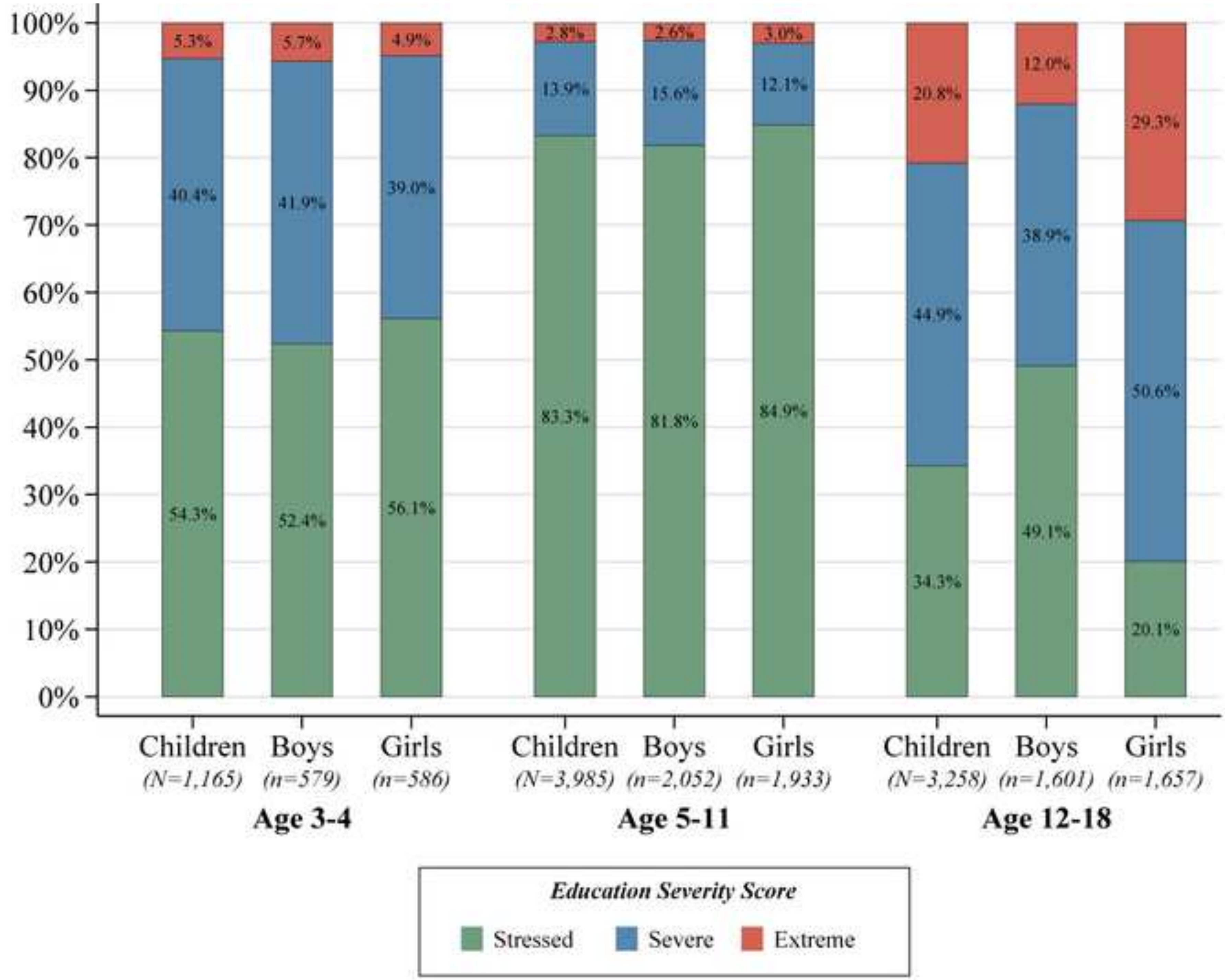

Fig 1B

Click here to access/download;Figure;Fig 1B.tif

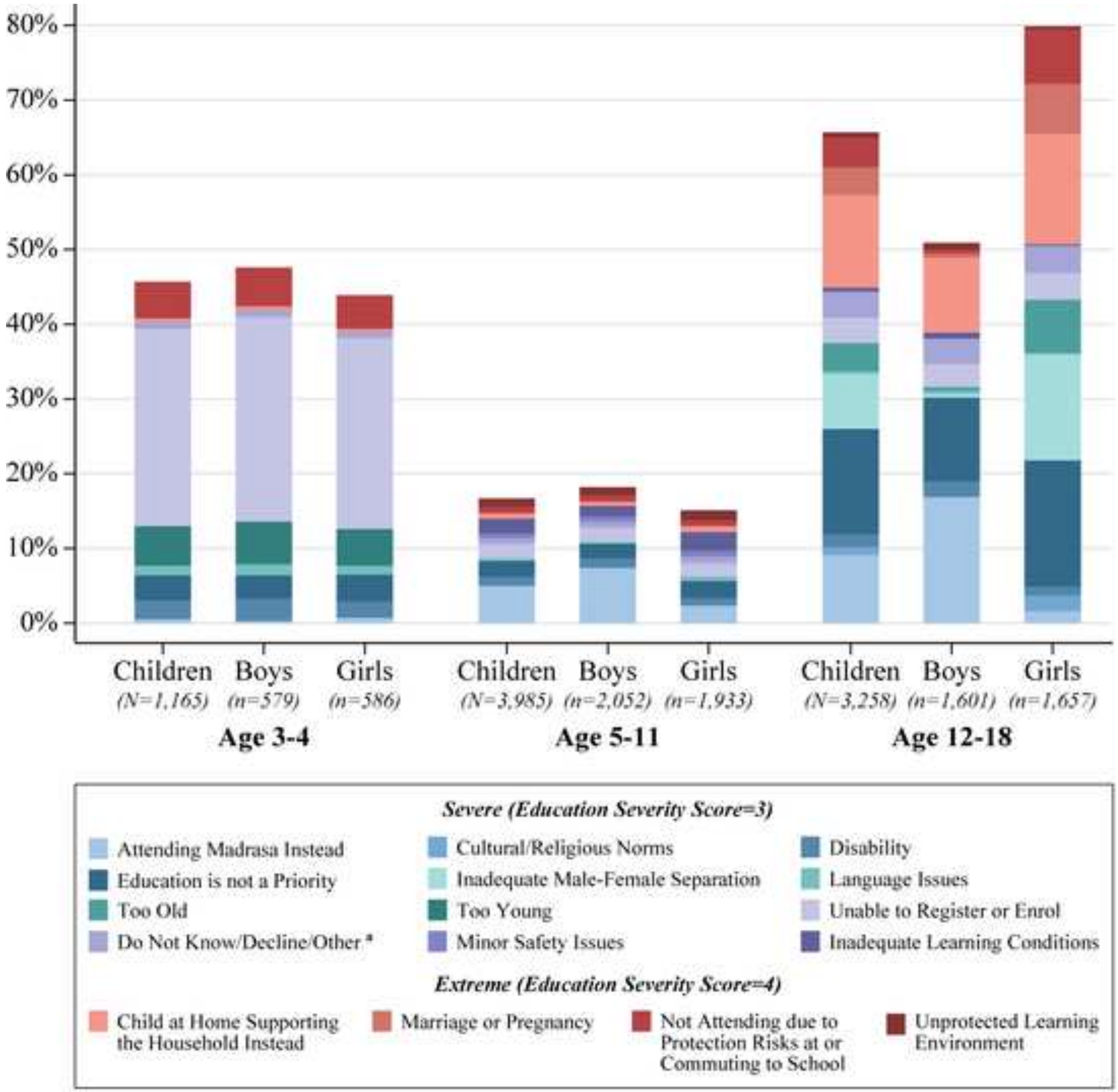

Fig 2A

Click here to access/download;Figure;Fig 2A.tif

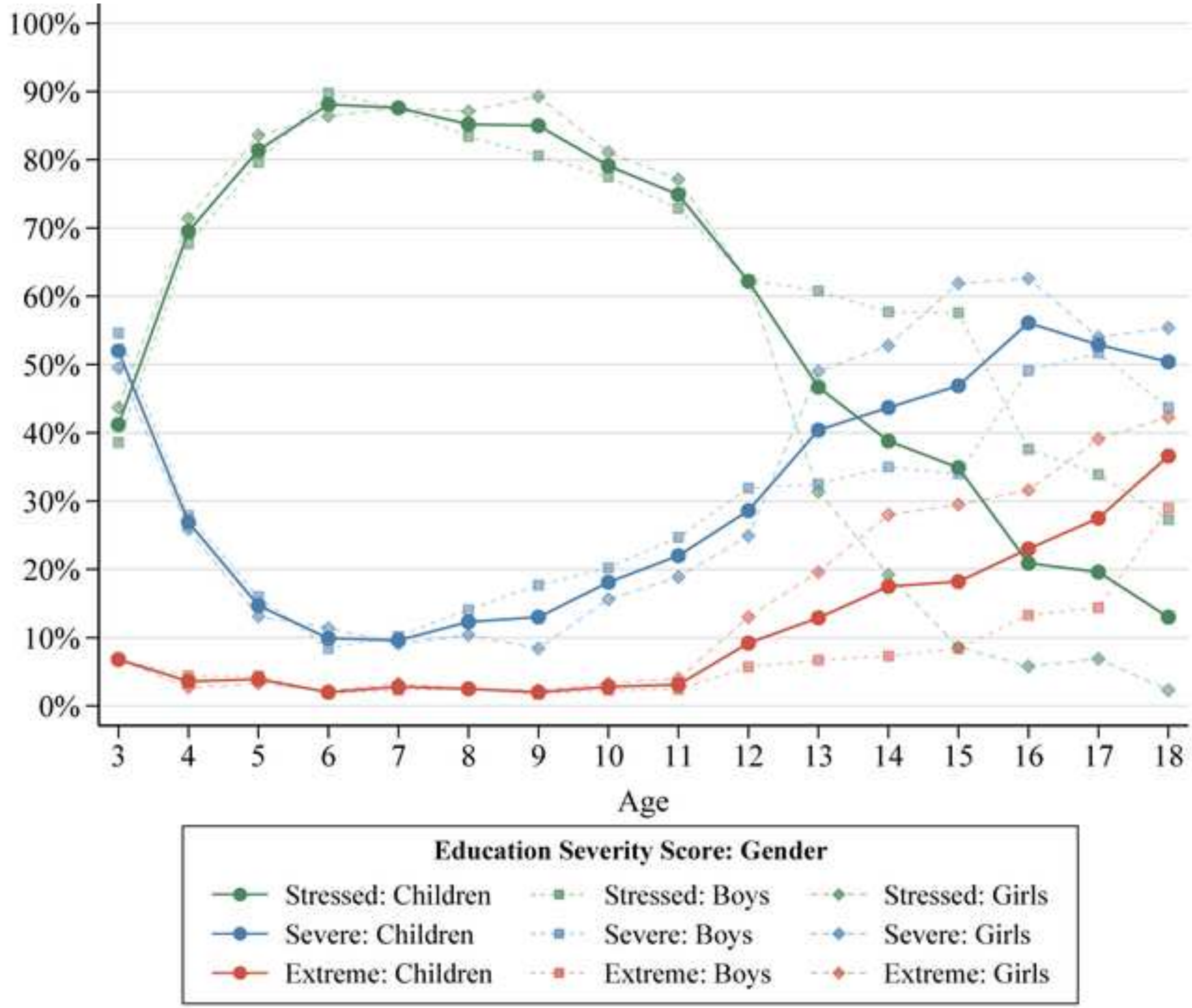

Fig 2B

Click here to access/download;Figure;Fig 2B.tif

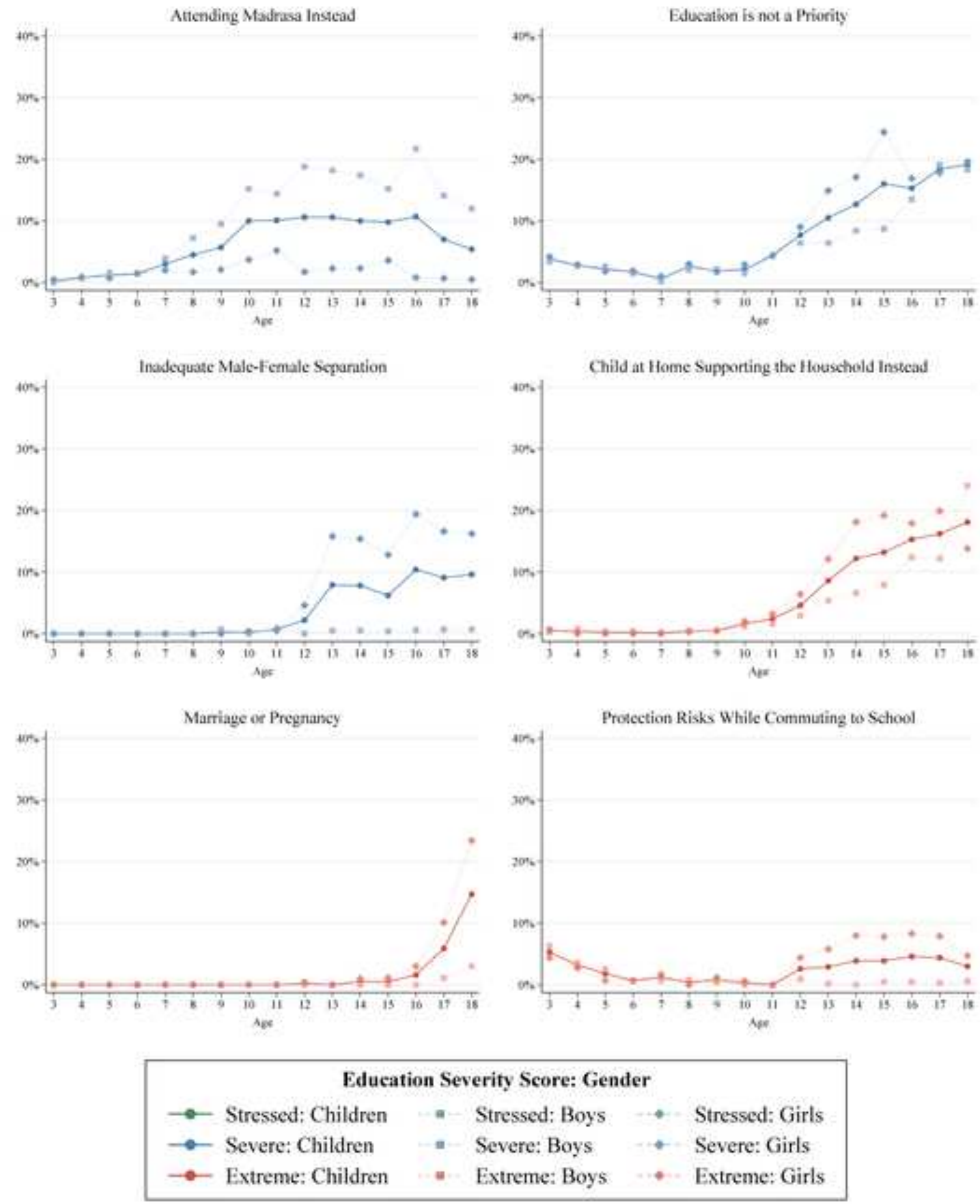

S1 Document

Click here to access/download
Supporting Information
S1 Document.docx

Prevalence of educational needs and factors associated with educational severity among Rohingya refugee children in Cox's Bazar, Bangladesh: a representative cross-sectional household survey

Harry J Wilson[1*], Nyanhial Yang[2], Md Nazmul Karim[1]
[1] School of Public Health and Preventive Medicine, Monash University, Melbourne, Victoria, Australia
[2] Murdoch Children's Research Institute, The Royal Children's Hospital, Melbourne, Victoria, Australia
[*] Corresponding author
Harry.J.Wilson@UNSWalumni.com

Abstract

Background

Approximately 1.2 million Rohingya refugees currently reside among the 33 refugee camps in Cox's Bazar - Bangladesh, where the right to education remains a critical humanitarian concern. This study aimed to estimate the prevalence of Rohingya refugee children in educational need, elucidate predominant reasons among children in need, and identify factors associated with educational severity to inform targeted humanitarian relief efforts.

Methods

This study utilized participant data collected during the 2023 Joint Multi-Sectoral Needs Assessment - a representative cross-sectional household survey. Education-age children (3-18) were assigned a severity score categorized as stressed, severe, or extreme based on proxy responses capturing educational access, learning conditions, and protection factors. Ordinal logistic regression examined factors associated with increasing educational severity scores.

Results

Among 8,408 education age children surveyed from 2,959 households, 29.6% were in severe educational need and 10.1% were in extreme educational need. After adjustment, increasing educational severity scores were significantly associated with head of household age 45-59 (aOR= 1.29 [95%CI: 1.09–1.54]), head of household age 60+ (aOR= 1.31 [95%CI: 1.07–1.60]), female headed households (aOR= 1.33 [95%CI: 1.15–1.54]), early education age boys (aOR= 3.95 [95%CI: 3.21–4.85]) and teenage boys (aOR= 4.42 [95%CI: 3.73–5.24]) relative to primary school age boys, early education age girls (aOR= 4.23 [95%CI: 3.36–5.31]) and teenage girls (aOR= 19.06 [95%CI: 15.70–23.15]) relative to primary school age girls, primary school age girls (aOR= 0.82 [95%CI: 0.68–0.99]) relative to primary school age boys, and teenage girls (aOR= 3.54 [95%CI: 3.04–4.12]) relative to teenage boys.

Conclusion

Educational needs remain highly prevalent among Rohingya refugee children – particularly teenage girls. Local humanitarian authorities are currently providing targeted relief to those in educational need but these interventions are threatened by sweeping international funding cuts. Future longitudinal studies are required to monitor educational needs over time and further clarify causal pathways.

Keywords: Rohingya; Humanitarian Emergency; Refugee; Refugee Camps; Human Rights;

Introduction

Eight years on from the mass displacement events in late 2017, approximately 1.2 million Rohingya refugees are currently sheltering within 33 congested camps of Cox's Bazar near the Bangladesh-Myanmar border [1].. The protracted nature of the Rohingya refugee crisis underscores the necessary operational transition from an acute emergency response to an enduring humanitarian program. Meeting the humanitarian needs of Rohingya refugees remains imperative for the international community but has met a critical juncture characterised by entrenched vulnerabilities, aid dependencies, concurrent domestic needs to support host communities, deteriorating humanitarian conditions within the camps, and a resurgence in targeted violence across the broader in Myanmar that has displaced an additional 150,000 Rohingya [1-7]. Although the contemporary humanitarian scenario has worsened, the international attention to the protracted crisis has waned [1,2,4,6,7]. The timing of these operational challenges precipitate amid a backdrop of changing geopolitical landscapes and receding humanitarian budgets that continues to threaten the delivery of essential humanitarian relief services for Rohingya refugees [1,3,5,8-10].

Approximately half of all Rohingya refugees sheltering among the camps in Cox's Bazar are education age children [1].. Consequentially, the demographic profile positions the education sector as a key cornerstone of the contemporary humanitarian response [1,5,10]. Beyond the direct individual benefits, ensuring the right to education is of paramount importance to protect vulnerable children, foster community resilience, build capacity for self-determination and strengthen prospective recovery efforts when circumstances allow safe voluntary repatriation [5,6,11-13]. A growing body of evidence highlights the pragmatic need for standardized education in emergency programs to incorporate nuanced age-gender responsive interventions that are co-designed with refugee communities, contextually framed within the lived experience of education age children they serve, and commensurate with the specific barriers they endure [14-19]. Additionally, the transition from childhood to adolescence may intensify gendered effects among school-age boys and girls that may differently interact with standardized education services [13,19,20]. This developmental period is particularly crucial for teenage girls, significantly influencing their physical, psychosocial, and emotional well-being that persist into adulthood [12,18]. Reciprocally, the implications of compromised educational attainment are compounded in protracted displacement settings such as the Rohingya refugee crisis where cumulative exposure over time threatens to not only widen but entrench gendered inequities [11,13,18,21].

Commented [HW1]: reference

Concerningly, prior analysis has identified the education sector as the leading reason for extreme humanitarian needs among Rohingya refugee households surveyed during the 2023 Joint Multi-Sectoral Needs Assessment (J-MSNA) [22]. Preliminary investigation foreshadowed gendered disparities and raised serious child protection concerns [22]. However, this analysis was restricted to extreme

educational needs only and did not disaggregate education sector severity scores by age, gender or underlying reasons that limits its ability to strategically inform sectoral programming [22].

Appling a more sensitive sector-specific investigation of the education sector using the same 2023 J-MSNA dataset, this study aimed to estimate the prevalence of Rohingya refugee children in educational need, evaluate the distribution of education sector severity by age, gender and attributable reason to intelligently inform specific interventions, and identify household vulnerability factors and demographic characteristics associated with educational severity scores to strengthen targeted relief efforts by local humanitarian authorities [23].

Methods

Study design, population and sampling strategy

This study draws upon anonymized participant data from the 2023 J-MSNA, a representative cross-sectional survey of Rohingya households within the 33 refugee camps of Cox's Bazar, used for secondary analysis [23]. The primary data collection was conducted by the REACH Initiative in conjunction with sector-specific authorities and local enumerators. The dataset was accessed on the 27th of March 2025 under license approved by UNHCR. The study population included all Rohingya refugee households registered within the 33 camps of Cox's Bazar [24,25]. An administratively complete sampling frame of registered households was compiled by camp authorities [24,25]. A total of 3,465 households were selected for data collection using stratified random sampling with camps as strata [24,25]. The number of households selected for sampling at each camp were guided by a single proportion sample size calculation that derived the minimum number of households needed to ensure camp-specific results could be concluded with a minimum level of precision (10% margin of error) at a 95% confidence level [24,25]. The sample size was adjusted for the finite household population that were eligible for selection at each camp and inflated by an additional 10% to mitigate non-response [24,25]. Although the study was primarily powered to enable camp specific results at the household-level, the achieved sample size provided sufficient precision for individual-level subgroup analyses by age and gender. Of the 3,465 households selected for sampling, a total sample size of 3,400 households were surveyed between August 27th and September 17th 2023 with individual data pertaining to 18,172 household members. Missingness was minimal (<1%) and these records were excluded from analysis.

Data collection

Data were collected through in-person household interviews administered by trained enumerators using a validated structured questionnaire that captured standardized humanitarian indicators and sector-specific items [24,25]. Gender-balanced field teams (comprising male and female interviewers) conducted interviews with an adult (age 18 or older) respondent of the same gender (self-identified)

[24,25]. Education status and reported reasons for educational need were provided by the adult respondent on behalf of each child within the household (proxy respondent), rather than by the children themselves. Responses were digitally recorded using the KoBo Collect application on mobile devices and routinely uploaded to a secure UNHCR server [24,25]. Raw participant data was cleaned according to the REACH Initiative's minimum standards protocol with a final de-identified dataset provided for statistical analysis [24,25]. Verbal informed consent was secured from all household respondents and formally recorded in the deidentified dataset. Respondents were informed of the survey's objectives, the voluntary nature of their participation, measures to protect response confidentiality and prerogative to decline any question or discontinue the interview at any time. Enumerators were educated on the principles of participant safeguarding and trained to provide formal referral protocols upon discovery of urgent protection or health concerns.

Variables

The primary outcome was education severity score assigned to each education age child (age 3-18) based on proxy responses ~~responses~~ to questionnaire items provided by an adult respondent on behalf of each education age child within the household that captured the four dimensions of educational access, learning conditions, protection and aggravating circumstances. Education severity scores were assigned based on recommended criteria outlined in the Global Education Cluster (UNICEF and Save the Children) severity classification manual, Joint Intersectoral Analysis Framework (JIAF) and Multi-Sectoral Needs Index (MSNI) [24,26,27]. Severity was scored according to the JIAF five-point intersectoral severity classification scale ranging from minimal (score=1) to catastrophic (score=5). The five-point intersectoral severity scale was operationalised as a three-point scale in line with J-MSNA methods due to contextual factors and sampling units rather than inferential constraints that may otherwise compromise the construct validity of the established classification scheme.

The minimal severity category (score=1) could not be assigned due to the absence of formal education services within the camps. Hence, the lowest possible score that could be assigned reflected regular attendance at a non-formal learning centre corresponding to a severity score of stressed (score=2) rather than minimal (score=1). Conversely, the catastrophic severity score (score=5) requires aggregate response-level data indicating complete sectoral collapse and systemic exclusion rather than individual learning conditions and reasons for not accessing education as reported by proxy respondents on behalf of the household.

The operationalized three-point scale categorised each education age child as either stressed (score=2), severe (score=3) or extreme (score=4). The restricted three-point scale retains the same ordinal severity distinctions of the established five-point intersectoral severity scale, supporting meaningful comparisons of educational severity scores across subgroups. The lowest possible score of stressed

(score=2) was defined as accessing non-formal education in a protected environment offering acceptable learning conditions.

Severe (score=3) was classified as either not accessing education due to non-aggravating circumstances, or accessing non-formal education in a protected environment but experiencing ~~poor~~ inadequate learning conditions or minor safety issues that do not present an immediate threat to the child's wellbeing. Inadequate learning conditions included curriculum is not adapted, displacement induced change, lack of qualified teachers, lack of teaching/learning material, language barriers, overcrowding, and poor water/sanitation/hygiene conditions. Minor safety issues included physical or verbal bullying between students, physical punishment from teachers, teaching staff unqualified in psychosocial support, and unsafe infrastructure

Extreme (score=4) was defined as either not accessing education services due to aggravating circumstances, or attending an unprotected learning environment due to serious safety issues that endanger the child's well-being. Aggravating circumstances correspond to the most severe accessibility barriers such as marriage or pregnancy, child paid and unpaid labour to support the household instead of attending school, recruitment by armed groups, discrimination, and safety threats whilst travelling to or attending school [24,27]. Unprotected learning environment included armed group recruitment, school attacks, gender based or sexual violence, and discrimination based on the child's ethnic, gender or religious identity [26,27]. The underlying reason for each child's education severity score were utilised as secondary outcomes to evaluate predominate underlying reasons for educational severity scores and their distribution across age-gender subgroups.

Individual characteristics of education age children and household vulnerability factors evaluated for association with educational needs were identified from previous J-MSNA research, Refugee Influx Emergency Vulnerability Assessments (REVA), and funding impact analysis conducted by the World Food Programme [8,9,28,29]. Age groups were assigned according to previous camp levels findings and appropriate cutoff points identified from trends observed over increasing age [30]. Subgroups among education age children (age 3-18) included early education age children (age 3-4), primary school age children (age 5-11) and teenage children (age 12-18). The rationale for the selected age cutoff point used to distinguish primary school age children (age 5-11) from teenage children (age 12-18) was based on mitigating contamination of age-groups from effect heterogeneity observed during linear trend analysis. -Head of household age was categorized into 15-year age groups. Marital status was collapsed into a binary variable that included married and single participants (no partner/separated/divorced/widowed) due to limited observations. Head of household disability status was classified as a binary variable corresponding to participants citing either "a lot of difficulty" or "cannot do it at all" to any of the six functional domains as per the Washington Group Short Set (WG-SS) guidelines [31]. Monthly household income per capita (household members) was categorized according to quartiles and utilized to investigate associations with financial vulnerability.

Statistical analysis

All results were computed using Stata version 19.5 survey functions that accounted for the stratified sampling design, adjusted for the clustering of education age children within the same household and controlled for differential probability of household selection between the camps (strata) using survey weights computed as the inverse probability of selection [32].

Descriptive statistics were used to analyse demographic characteristics of sampled households and education age children. Weighted frequencies and percentages were reported for categorical variables. Median and interquartile range (IQR) or mean and standard deviation (SD) were reported for continuous variables depending on symmetry. Prevalence estimates of children and households experiencing educational needs were reported as weighted percentages with 95% confidence intervals (95%CIs) for precision.

Education severity scores and their attributable reasons were disaggregated by age and gender. The distribution of severity scores and reported reasons were assessed over increasing age and by gender to assess linear trends. Ordinal logistic regression analysis was conducted to investigate factors associated with increasing education sector severity scores and the assumption of proportional odds was tested. Unadjusted odds ratios (OR) and adjusted odds ratios (aOR) were reported for bivariate and multivariate estimates respectively. A threshold value of $p \leq 0.05$ was used to conclude statistical significance and 95%CIs were reported for precision using design-adjusted standard errors computed via Taylor series linearization.

Potential effect modification was considered between head of household gender and marital status to investigate concentrated effects among single female headed households. Two-way age and gender interactions among education age children (age-gender and gender-age) were investigated using both stratified analysis and interaction terms. Gender-age interactions were investigated using stratified (by gender) analysis reporting the relative risk associated with age groups among boys and among girls. Age-gender interactions were investigated using interaction terms to evaluate the relative risk associated with girls relative to boys within the same age group. Pairwise correlation and variance inflation factor (VIF) analysis were conducted to evaluate potential problematic multicollinearity.

Results

Participant characteristics

Among the 3,400 total households sampled, 441 households did not contain education age children (age 3-18) and were excluded from subsequent analysis (Table 1). A total of 8,408 education age children were surveyed from the remaining 2,959 households across the 33 camps. The number of households without education age children (n=441) did not significantly differ between the camps ($p > 0.05$). The

proportional contribution of each camp to the overall sample showed minimal variation (mean change= +0.5%; range: -12.6% to +8.7%) before (N=3,400) and after (N=2,959) the exclusion of households without education age children. The number of households and education age children sampled per camp was approximately normally distributed with a mean of 90 households (SD= 35; Table 1) and 255 education age children (SD= 95; Table 1) respectively. The median head of household age was 39 years (IQR: 30 – 50; Table 1) and the majority of households were male headed (79.8%; Table 1). Approximately half of surveyed children were aged between 5-11 years (47.4%; Table 1) and the proportion of boys sampled (50.3%; Table 1) was slightly higher than the proportion of girls (49.7%; Table 1). Less than two-thirds of education age children were enrolled and regularly attending informal education within the camps (60.3%; Table 1). The prevalence of children in educational need was 39.7% (95% CI: 38.4% – 41.0%) and almost two-thirds of households contained at least one child in educational need (65.7% [95% CI: 63.8% – 67.5%]).

Table 1. Characteristics of sampled households (N= 2,959) and education age children (N= 8,408)

	**Summary***
**Households per Camp, Mean (N= 2,959)**	90 (SD: 35)
**Education Age Children per Camp, Mean (N= 8,408)**	255 (SD: 95)
**Head of Household Age, Median (N= 2,959)**	39 [IQR: 30 – 50]
18-29	688 (23.2%)
30-49	1414 (47.8%)
50+	857 (29.0%)
**Head of Household Gender (N= 2,959)**	
Male	2361 (79.8%)
Female	598 (20.2%)
**Education Age Children per Household, Median (N= 2,959)**	3 [IQR: 2 – 4]
1-2	1465 (49.5%)
3-5	1284 (43.4%)
6-9	210 (7.1%)
**Age, median (N= 8,408)**	10 [IQR: 6 – 14]
3-4	1,165 (13.9%)
5-11	3,985 (47.4%)
12-18	3,258 (38.7%)
**Gender (N= 8,408)**	
Boys	4233 (50.3%)
Girls	4175 (49.7%)
**Education Sector Severity (N= 8,408)**	
Stressed (Severity Score=2)	5070 (60.3%)
Severe (Severity Score=3)	2488 (29.6%)
Extreme (Severity Score=4)	850 (10.1%)
**Prevalence of Educational Need[a]**	
Children in Educational Need (N= 8,408)	39.7% [95% CI: 38.4% – 41.0%]
Households in Educational Need (N= 2,959)	65.7% [95% CI: 63.8% – 67.5%]

* Estimates are relative to either the total number of households with education age children (N= 2,959) or the total number of education age children (N= 8,408). Summary statistics are weighted frequency and weighted percentages for categorical variables, weighted median (IQR) for asymmetrically distributed continuous variables, weighted mean (SD) for symmetrical continuous variables, or weighted population prevalence (%) and 95% CIs for outcome variables.

[a] Children in educational need was classified as severe or extreme education severity scores (severity score=3+) as per MSNI methods [24]. Children in education need defined as per JIAF and education cluster guidelines - Education age children in the areas affected by crisis who do not have access to protective education and acceptable learning conditions, which can negatively impact (i) their physical and psychosocial wellbeing, (ii) cognitive development, and (iii) their ability to meet their future needs [26,27]. Households in educational need categorised as those with at least one child in educational need as per MSNI methods [24].

Educational Severity by age and gender

The distribution of education sector severity scores (Fig 1A) and their attributable reasons (Fig 1B) disaggregated by age-group and gender demonstrated three clear patterns: (i) substantial unmet educational need in early childhood driven by enrolment barriers, (ii) high attendance among children aged 5–11 with limited extreme need, and (iii) a sharp decline in enrolment after age 12 with pronounced gender disparities. Approximately half (54.3%; Fig 1A) of early education age children (3-4 years) were enrolled and regularly attending informal education with similar results observed among boys (52.4%) and girls (56.1%). ~~An additional 40.4% were found to be in severe educational need mostly attributable to “unable to register or enrol” (26.5%; Fig 1B), “too young” (5.3%; Fig 1B) and “education is not a priority” (3.3%; Fig 1B).~~ A ~~further~~ small proportion~~5.3%~~ of early education age children were found to be experiencing extreme educational need mostly attributable to “protection

risks while commuting to school" (4.3%; Fig 1B). Most primary school age children (5-11) were enrolled and regularly attending informal education (83.3%; Fig 1A) with similar results observed between boys (81.8%; Fig 1A) and girls (84.9%; Fig 1A). Comparatively, An estimated 13.9% of primary school age children (5-11) were in severe educational need predominantly attributable to "attending madrasa instead" (4.9%; Fig 1B), especially among boys (7.4%; Fig 1B) compared to girls (2.3%; Fig 1B). A relatively small proportion of primary school age children (5-11) were found to be in extreme educational need (2.8%; Fig 1A). only Only a third (34.4%; Fig 1A) of teenage children age 12-18 were enrolled and regularly attending informal education with significant heterogeneity observed between boys (49.1%; Fig 1A) and girls (20.1%; Fig 1A). Most teenage children were experiencing severe educational needs (44.9%; Fig 1A) with greater prevalence observed among teenage girls (50.6%; Fig 1A) compared to boys (38.9%; Fig 1A).

Fig 1A. Education severity scores among education age children by age group and gender.

Fig 1B. Attributable reason for educational needs among children (age 3-18) by age group and gender.

[a] Rarely reported reasons (<1%) for severe educational needs among all cohorts were recoded as "other" rather than individually categorised for visual clarity. "Other" includes the following reasons and percentages:
Children Age 3-4: Lack of Schools (0.3%), Language Issues (1.3%), Decline/Do not Know (0.6%)
Boys Age 3-4: Lack of Schools (0.2%), Language Issues (1.5%), Decline/Do not Know (0.7%)
Girls Age 3-4: Lack of Schools (0.4%), Language Issues (1.2%), Decline/Do not Know (0.4%)
Children Age 5-11: Illness (0.1%), Lack of Schools (0.2%), Decline/Do not Know (0.5%)
Boys Age 5-11: Cannot Afford Education Costs (0.1%), Illness (0.1%), Lack of Schools (0.1%), Decline/Do not Know (0.5%)
Girls Age 5-11: Illness (0.1%), Lack of Schools (0.4%), Decline/Do not Know (0.4%)
Children Age 12-18: Cannot Afford Education Costs (0.2%), Child Uninterested (0.3%), Home Based Learning (0.4%), Inadequate Curriculum (0.2%), Decline/Do not Know (2.4%)
Boys Age 12-18: Cannot Afford Education Costs (0.3%), Child Uninterested (0.5%), Home Based Learning (0.7%), Inadequate Curriculum (0.1%), Decline/Do not Know (1.8%)
Girls Age 12-18: Cannot Afford Education Costs (0.1%), Child Uninterested (0.1%), Home Based Learning (0.1%), Illness (0.1%), Inadequate Curriculum (0.2%), Decline/Do not Know (3.0%)

The analysis of education severity scores over increasing age and by gender illustrated ~~that~~ similar severity profiles among boys and girls ~~were similar~~ between ages 3-11, followed by a ~~(Fig 2A). An inverse proportional relationship was also evident between stressed and severe education severity scores with a steady proportion of children age 3-11 experiencing extreme educational needs. A~~ sharp decline in the proportion of children enrolled and regularly attending school ~~was observed over~~among teenagers (ages 12-18) with substantial ~~, g~~Gender~~ed~~ disparities (Fig 2A). ~~regarding severe and extreme educational needs emerged between ages 11-12 (Fig 2A).~~ A rapid rise in severe educational needs was observed among adolescent girls age 12-15 (Fig 2A), predominantly attributable to “education is not a priority” and “inadequate male-female separation” ~~as reported by household respondents~~ (Fig 2B). A linear trend between extreme educational needs and increasing age was evident among teenage girls age 11-18, ~~(Fig 2A). The increasing trend in the proportion of girls~~mainly attributable to ~~age 11-15 experiencing extreme educational need was mostly attributable to~~ “child at home supporting the household instead”, and not attending due to “protection risks at or commuting to school” during ages 12-15, followed by an exponential rise ~~(Fig 2B). In contrast, the continuous rise~~ in ~~extreme educational needs among girls age 16-18 was primarily due to~~ “marriage or pregnancy” among teenage girls age 16-18 ~~-~~(Fig 2B). Educational needs among teenage boys remained relatively constant until the ages of 16-18 ~~between age 12-15 then increased between 16-18~~ predominantly attributable to “education is not a priority” (Fig 2B) and “child at home supporting the household instead” (Fig 2B).

Fig 2A. Education severity scores over increasing age (3-18) and by gender (children, boys, girls).

Fig 2B. Reported reason for educational needs over increasing age (3-18) and by gender (children, boys, girls).

Household vulnerability factors and individual characteristics associated with educational severity

In unadjusted analysis, increasing educational severity scores were associated with older head of households, female headed households, head of households living with a disability, age-groups among boys, age-groups among girls, and girls relative to boys within the same age group.

After adjustment in multivariable analysis, increasing educational severity scores were significantly associated with head of household age 45-59 (aOR= 1.29 [95%CI: 1.09–1.54]), head of household age 60+ (aOR= 1.31 [95%CI: 1.07–1.60]), female headed households (aOR= 1.33 [95%CI: 1.15–1.54]), early education age boys (aOR= 3.95 [95%CI: 3.21–4.85]) and teenage boys (aOR= 4.42 [95%CI: 3.73–5.24]) relative to primary school age boys, early education age girls (aOR= 4.23 [95%CI: 3.36–5.31]) and teenage girls (aOR= 19.06 [95%CI: 15.70–23.15]) relative to primary school age girls, primary school age girls (aOR= 0.82 [95%CI: 0.68–0.99]) relative to primary school age boys, and teenage girls (aOR= 3.54 [95%CI: 3.04–4.12]) relative to teenage boys

The multivariate model indicated adequate fit over a null model ($p<0.001$) and did not violate the assumption of proportional odds. No evidence of problematic multicollinearity was observed, all pairwise correlations between independent variables were below 0.7 and the mean VIF was 1.5 with no extreme outliers ($VIF_{max}<10$). No statistically significant evidence of an interaction on the multiplicative scale was found between female headed households and marital status ($p>0.05$) with main effects reported in consequence.

Table 2. Individual factors and household characteristics associated with higher educational severity scores.

	Summary	Odds Associated with Higher Educational Severity Scores			
	(N=8,408)	Unadjusted		Adjusted	
	n (%)	OR (95%CI)	p-value	aOR (95%CI)	p-value
**Head of Household Age**					
18-34	2,521 (30.0%)	1.00	–	1.00	–
35-44	2,577 (30.6%)	**1.28 (1.10–1.47)**	**<0.001***	0.92 (0.77–1.09)	0.319
45-59	2,225 (26.5%)	**2.39 (2.08–2.75)**	**<0.001***	**1.29 (1.09–1.54)**	**0.003***
60+	1,085 (12.9%)	**2.80 (2.36–3.31)**	**<0.001***	**1.31 (1.07–1.60)**	**0.008***
**Head of Household Gender**					
Male	6,925 (82.4%)	1.00	–	1.00	–
Female	1,483 (17.6%)	**1.50 (1.31–1.72)**	**<0.001***	**1.33 (1.15–1.54)**	**<0.001***
Female: Married[a]	780 (9.3%)	1.00	–	1.00	–
Female: Single/Divorced/Widowed[a]	703 (8.3%)	1.15 (0.91–1.46)	0.241	1.01 (0.78–1.30)	0.960
**Head of Household Disability Status**					
No Disability	6,972 (82.9%)	1.00	–	1.00	–
Living with a Disability	1,436 (17.1%)	**1.19 (1.03–1.37)**	**0.017***	1.03 (0.88–1.21)	0.686
**Monthly Household Income (per capita)**[b]					
1st Quartile (<$US 12.5)	2,385 (28.4%)	1.00	–	1.00	–
2nd Quartile ($US 12.5–17.49)	2,379 (28.3%)	0.99 (0.86–1.14)	0.903	1.09 (0.93–1.28)	0.280
3rd Quartile ($US 17.5–24.99)	2,174 (25.9%)	0.90 (0.77–1.04)	0.148	0.95 (0.81–1.12)	0.543
4th Quartile (≥$US 25)	1,470 (17.4%)	1.08 (0.92–1.26)	0.363	1.13 (0.95–1.35)	0.157
**Boys: Age Group**					
Boys: Age 5-11	2,053 (24.4%)	1.00	–	1.00	–
Boys: Age 3-4	579 (6.9%)	**3.84 (3.14–4.70)**	**<0.001***	**3.95 (3.21–4.85)**	**<0.001***
Boys: Age 12-18	1,601 (19%)	**4.82 (4.09–5.68)**	**<0.001***	**4.42 (3.73–5.24)**	**<0.001***
**Girls: Age Group**					
Girls: Age 5-11	1,933 (23%)	1.00	–	1.00	–
Girls: Age: 3-4	585 (7%)	**4.12 (3.30–5.14)**	**<0.001***	**4.23 (3.36–5.31)**	**<0.001***
Girls: Age 12-18	1,657 (19.7%)	**21.23 (17.56–25.67)**	**<0.001***	**19.06 (15.70–23.15)**	**<0.001***
**Age 3-4: Gender**					
Age 3-4: Boys	579 (6.9%)	1.00	–	1.00	–
Age 3-4: Girls	585 (7%)	0.87 (0.70–1.09)	0.221	0.88 (0.70–1.10)	0.263
**Age 5-11: Gender**					
Age 5-11: Boys	2,053 (24.4%)	1.00	–	1.00	–
Age 5-11: Girls	1,933 (23%)	**0.81 (0.67–0.98)**	**0.033***	**0.82 (0.68–0.99)**	**0.044***
**Age 12-18: Gender**					
Age 12-18: Boys	1,601 (19%)	1.00	–	1.00	–
Age 12-18: Girls	1,657 (19.7%)	**3.58 (3.07–4.17)**	**<0.001***	**3.54 (3.04–4.12)**	**<0.001***

* Statistically significant at p ≤ 0.05.

[a] Additional estimates from a duplicate model with interaction terms applied to investigate effect modification among single/divorced/widowed female headed households relative to married female headed households. Note that estimates reported for female headed households represent the main effects among all female headed households rather than the subgroup of married female headed households. All multivariable estimates were adjusted for main effects only.

[b] Monetary value in 2023 US dollars.

Discussion

The demographic characteristics of households with education age children closely mirror those of all sampled households that prior analysis demonstrated to be strongly aligned with UNHCR's database of all Rohingya refugees registered within the camps [22]. The alignment observed between the sample and population characteristics strongly support the representativeness of participants and hence generalisability of our results [22].

Despite ongoing humanitarian relief efforts by local authorities, the findings highlight that almost two-thirds of households, and 2-in-every-5 children age 3-18 were in educational need. Disaggregation by age group illustrated the higher prevalence of educational needs among early education age children (age 3-4) and teenage children (age 12-18). Further disaggregation by gender evidenced the similarities among boys and girls during childhood (age 3-11), followed by the rapid divergence of educational needs during adolescence (age 12-18). These age-gender dynamics are best illustrated by the distribution of severity scores and their attributable reasons over increasing age that further evidenced the gendered similarities during childhood and contrasting linear trends observed during adolescence that may reflect the emergence of gendered interactions that differentiate teenage boys and teenage girls. These descriptive interpretations are supported by the logistic regression results that revealed a sharp increase in the odds associated with teenage girls relative to both primary school age girls and teenage boys.

The associations observed between educational needs and individual age-gender characteristics persisted with limited attenuation after adjustment for common household vulnerability factors. The comparative strength of association with individual-level severity scores among education age children may suggest the broader influence of exogenous structural factors and systemic challenges within the education sector such as restrictive enrolment policies experienced by teenage children that may provoke the increasing trend in educational severity scores observed from age 11 onwards. This interpretation regarding exogenous structural constraints are ~~is~~ consistent with findings from previous sector-specific analysis that particularly highlight the limited learning opportunities for teenage girls ~~and lack of reintegration support for out-of-school teenagers transitioning from at-home to face-to-face learning as COVID-19 restrictions subsided~~ [5,14,15,21,28]. Similarly, our findings align with education-in-emergency frameworks that conceptualise schooling as both an important humanitarian service and protective system, with an emphasis on safeguarding the heightened vulnerability among adolescent children. In contrast, the elevated ~~risk~~ odds associated with female headed households, young adult (18-24) headed households and elderly (age 45+) headed households may suggest the influence ~~draw focus towards the~~ of survival pressures that ~~may~~ could potentially provoke households to deprioritise their children’s education. These interpretations are supported by the distribution of reported reasons for educational need, which are highly concentrated among teenage girls. The trends regarding deprioritization and deteriorating severity scores highlight the plausible intersections between

the education sector and the broader survival pressures within the camps that may position households to adopt negative livelihood coping strategies [33,34]. In addition, the high proportion of teenage girls in extreme educational need reportedly due to childhood marriage and early pregnancy emphasize the close links between deteriorating educational needs and serious child protection issues that require urgent remediation efforts.

Limitations

The findings should be interpreted within the context of multiple strengthens and limitations. The stratified random sampling, survey weighting and face-to-face interviews enhance representativeness of participants. However, the findings are predominantly limited by the use of self-reported data collected from proxy respondents on behalf of the household that may consequentially introduce measurement bias and undermine the accuracy of the results.

The cross-sectional design limits causal inference and observed associations may reflect unmeasured confounding such as . These potential sources of confounding includingcomplex developmental issues that may modulate individual abilities to engage with the education sector. Additionally, several key determinants of educational access—such as learning-centre proximity, teacher availability, household mobility restrictions, and camp-level security conditions that were not directly measured.

Finally, sensitive protection-related barriers (e.g., marriage, pregnancy, exploitation, safety threats) may be under-reported due to stigma and proxy reporting, potentially leading to conservative estimates of extreme need and potentially lower odds ratios among girls that are more likely to experience both sensitivities and stigmatisation.

Implications for practice

Our findings present numerous implications for humanitarian programming within the education sector. The age- and gender-specific gradients identified in this study suggest that education programming should be consolidated among early education and primary school age children, and differentiated among teenage boys and teenage girls to strategically target gendered protection issues, sociocultural barriers and accessibility constraints. In response, over 20 humanitarian projects coordinated by lead agencies, international authorities and non-government organisations are currently providing relief to Rohingya refugee children in educational need [5]. These projects include school feeding programs for disadvantaged students, reuptake programs for out-of-school children, accelerated learning programs for students who are at risk of falling further behind and restructuring of learning centres to provide female-only classrooms that redress sociocultural barriers among teenage girls [4,10]. The continuity of these projects are critical to affirm the right to education among Rohingya refugee children yet remain desperately underfunded at just 15% of their stated requirements [35]. The detrimental impacts of funding cuts among the shock-prone education sector are already evident, provoking the closure of 43%

of learning facilities and denying access to education for approximately 200,000 Rohingya children [34,36]. Rapid restructuring efforts in conjunction with community adaptation have secured the gradual recovery of the education sector [34]. However, these gains remain fragile and unable to deliver the sustained relief that the protracted crisis and their multidimensional complexities demand [10,34].

Conclusion

Educational needs remain highly prevalent among Rohingya refugee children in Cox's Bazar. Approximately a third of children age 3-18 were found to be in severe educational need and one in ten were experiencing extreme educational need. The findings reveal pronounced age-gender dynamics with teenage girls experiencing markedly higher educational needs, consistent with reported barriers related to deprioritisation, household survival pressures, sociocultural constraints and serious child protection concerns. Addressing the educational needs of Rohingya refugee children remains imperative to prevent gendered inequities, foster community resilience and strengthen recovery prospects from the protected protracted crisis. Ameliorating the scale of children in educational need, their complexities and intersectoral dependencies requires strategic, intensive and sustained humanitarian relief efforts. Future longitudinal and qualitative research is needed to redress study design constraints, –closely monitor responses to educational needs over time, better capture sensitive protection-related barriers to education in consultation with community, and clarify causal pathways involving household vulnerability factors and individual age-gender characteristics that may act to influence individual educational severity scores., and better capture sensitive protection-related barriers to education in consultation with community.

Acknowledgements

The authors wish to formally acknowledge those whose collective efforts have made this research article possible. Specifically, the Inter-Sector Coordination Group (ISCG), UNHCR, IOM, ECHO, the REACH Initiative, enumerators, other study personnel, and household respondents who graciously donated their time.

References

1. Inter-Sector Coordination Group (ISCG). Recalibrating the Rohingya Response: 2025-26 Joint Response Plan - Rohingya Humanitarian Crisis, January 2026 - December 2026. 2026. Available from: https://reliefweb.int/report/bangladesh/recalibrating-rohingya-response-2025-26-joint-response-plan-rohingya-humanitarian-crisis.
2. United Nations Human Rights Council. Situation of human rights of Rohingya Muslims and other minorities in Myanmar. 2025. Available from: https://digitallibrary.un.org/record/4084836.
3. Médecins Sans Frontières. The illusion of choice: Rohingya voices echo from the camps. 2025. Available from: https://reliefweb.int/report/bangladesh/illusion-choice-rohingya-voices-echo-camps.

4. Inter-Sector Coordination Group (ISCG). Bangladesh: Rohingya Refugee Response - Flash Appeal for 150,000 New Arrivals (June - December 2025). 2025. Available from: https://reliefweb.int/report/bangladesh/bangladesh-rohingya-refugee-response-flash-appeal-150000-new-arrivals-june-december-2025.
5. Inter-Sector Coordination Group (ISCG). Bangladesh: Rohingya Humanitarian Crisis Joint Response Plan January 2025 - December 2026. 2025. Available from: https://reliefweb.int/report/bangladesh/bangladesh-rohingya-humanitarian-crisis-joint-response-plan-january-2025-december-2026.
6. United Nations General Assembly. Situation of human rights of Rohingya Muslims and other minorities in Myanmar: report of the United Nations High Commissioner for Human Rights. 2025. Available from: https://digitallibrary.un.org/record/4092194/files/A_HRC_60_20-EN.pdf.
7. Borak Z, Haque S. Rohingya refugees in Bangladesh should be allowed to work. Lancet Psychiatry. 2026;13(5). doi: 10.1016/s2215-0366(26)00055-6.
8. World Food Programme (WFP). Impact of Cuts - Bangladesh: Outcome of 2023 pilot study: Impact of funding shortfalls on beneficiaries. 2024. Available from: https://reliefweb.int/report/bangladesh/impact-cuts-bangladesh-outcome-2023-pilot-study-impact-funding-shortfalls-beneficiaries-august-2024.
9. The World Bank, World Food Programme (WFP). Impact of Food Ration Cuts on the Displaced Rohingya Population in Cox's Bazar. 2024. Available from: https://reliefweb.int/report/bangladesh/bangladesh-impact-food-ration-cuts-displaced-rohingya-population-coxs-bazar-may-2024.
10. Inter-Sector Coordination Group (ISCG). Rohingya Refugee Response Urgent priorities: Addressing the Most Pressing Needs of the Joint Response Plan. 2025. Available from: https://reliefweb.int/report/bangladesh/bangladesh-rohingya-refugee-response-urgent-priorities-addressing-most-pressing-needs-joint-response-plan-june-december-2025.
11. World Health Organization. World report on promoting the health of refugees and migrants: monitoring progress on the WHO global action plan. 2026. Available from: https://iris.who.int/handle/10665/384927.
12. World Health Organization. WHO guideline on preventing early pregnancy and poor reproductive outcomes among adolescents in low- and middle-income countries. 2025. Available from: https://iris.who.int/handle/10665/381100.
13. Das J, Prima MM, Rimu FH, Hossain P, Das T, Nesa F, et al. Mental Health and Wellbeing of Rohingya Refugees: A Scoping Review. Journal of Racial and Ethnic Health Disparities. 2026. doi: 10.1007/s40615-026-02982-4.
14. REACH. Assessment of the Education Sector response to the Rohingya crisis - Rohingya refugee response. 2021. Available from: https://reliefweb.int/report/bangladesh/bangladesh-assessment-education-sector-response-rohingya-crisis-rohingya-refugee.
15. Cox's Bazar Education Sector. Joint Education Needs Assessment: Rohingya Refugee in Cox's Bazar - June 2018. 2018. Available from: https://reliefweb.int/report/bangladesh/joint-education-needs-assessment-rohingya-refugee-cox-s-bazar-june-2018.
16. Education Cannot Wait. Holistic learning outcomes measurement handbook for education in emergencies and protracted crises. 2025. Available from: https://reliefweb.int/report/world/holistic-learning-outcomes-measurement-handbook-education-emergencies-and-protracted-crises.
17. Inter-agency Network for Education in Emergencies (INEE). INEE Minimum Standards for Education: Preparedness, Response, Recovery (2024 Edition). 2024. Available from: https://reliefweb.int/report/world/inee-minimum-standards-education-preparedness-response-recovery-2024-edition.
18. Inter-agency Network for Education in Emergencies. Mind the Gap 3: Equity and Inclusion in and through Girls' Education in Crisis. 2023. Available from: https://reliefweb.int/report/world/mind-gap-3-equity-and-inclusion-and-through-girls-education-crisis-enpt.
19. Hossain MA, Zablotska-Manos I. Family planning awareness and contraceptive use among young Rohingya refugee women: A representative cross-sectional study in Cox's Bazaar, Bangladesh. Womens Health (Lond). 2026;22. doi: 10.1177/17455057261416251.

20. Iiyama S, Izutsu T, Miyamoto Y, Yesmin S, Begum T, Mariam E, et al. Effectiveness of community-based mental health and psychosocial support through the Paracounsellor model for Rohingya refugees in Bangladesh. Public Health in Practice. 2026;11. doi: 10.1016/j.puhip.2026.100799.

21. Østby G, Gjerløw H, Karim S, Dunlop E. Left Further Behind after the COVID-19 School Closures: Survey Evidence on Rohingya Refugees and Host Communities in Bangladesh. Journal on Education in Emergencies. 2023;9(1). doi: 10.33682/a1zn-5nda.

22. Wilson HJ. Drivers and determinants of extreme humanitarian needs among Rohingya refugee households: Evidence from UNHCR's multi-sectoral needs analysis. PLOS ONE. 2025;20(12):e0331727. doi: 10.1371/journal.pone.0331727.

23. United Nations High Commissioner for Refugees (UNHCR), International Organization for Migration (IOM), Directorate-General for European Civil Protection and Humanitarian Aid Operations (ECHO). Bangladesh: Joint Multi Sector Needs Assessment: Cox's Bazar, Rohingya Refugee Response – 2023, Refugees. 2023. Available from: https://microdata.unhcr.org/index.php/catalog/1128.

24. REACH. Bangladesh J-MSNA - MSNI Methodological Overview. 2023 [cited 2025 Apr 4]. Available from: https://repository.impact-initiatives.org/document/impact/474ac9e5/BGD2301_2023-J-MSNA_-MSNI-Methodological-overview_Jan-2024.pdf.

25. REACH. Research Terms of Reference: Joint Multi-Sector Needs Assessment MSNA 2023, Bangladesh. 2023 [cited 2025 Apr 4]. Available from: https://reliefweb.int/report/bangladesh/research-terms-reference-joint-multi-sector-needs-assessment-msna-2023-bgd2301-bangladesh-august-2023-v-1.

26. United Nations Office for the Coordination of Humanitarian Affairs (UNOCHA). Joint and Intersectoral Analysis Framework (JIAF) 2 Technical Manual. 2024. Available from: https://reliefweb.int/report/world/joint-and-intersectoral-analysis-framework-jiaf-2-technical-manual-july-2024.

27. Global Education Cluster. Calculating Education Cluster People in Need (PiN). 2024. Available from: https://www.educationcluster.net/calculating-people-need.

28. Inter-Sector Coordination Group (ISCG). Joint Multi-Sector Needs Assessment (J-MSNA): Bangladesh Rohingya Refugees - December 2021. 2022. Available from: https://reliefweb.int/report/bangladesh/joint-multi-sector-needs-assessment-j-msna-bangladesh-rohingya-refugees-december-2021.

29. World Food Programme (WFP). Bangladesh: Refugee Influx Emergency Vulnerability Assessment (REVA-8) Report, June 2025. 2025. Available from: https://reliefweb.int/report/bangladesh/bangladesh-refugee-influx-emergency-vulnerability-assessment-reva-8-report-june-2025.

30. Inter-Sector Coordination Group (ISCG). Joint Multi-Sector Needs Assessments (J-MSNA) Factsheet - Refugee Camp-level findings - December. 2023. Available from: https://reliefweb.int/report/bangladesh/bangladesh-joint-multi-sector-needs-assessment-j-msna-camp-level-findings-december-2023.

31. The Washington Group on Disability Statistics. The Washington Group Short Set on Functioning (WG-SS). 2022 [cited 2025 Apr 4]. Available from: https://www.washingtongroup-disability.com/question-sets/wg-short-set-on-functioning-wg-ss/.

32. StataCorp. Survey Data Reference Manual. 2025. Available from: https://www.stata.com/bookstore/survey-data-reference-manual/.

33. World Food Programme (WFP). Livelihood Coping Strategies Indicator for Food Security: List of strategies and their definitions. 2023 [cited 2025 June 6]. Available from: https://docs.wfp.org/api/documents/WFP-0000147820/download/.

34. United Nations Office for the Coordination of Humanitarian Affairs (OCHA). Global Humanitarian Overview 2026: Rohingya Joint Response Plan (JRP). 2026 [cited 2026 Jan 8]. Available from: https://humanitarianaction.info/document/global-humanitarian-overview-2026/article/rohingya-joint-response-plan-jrp-1.

35. Inter-Sector Coordination Group (ISCG). Bangladesh: Rohingya Humanitarian Crisis Joint Response Plan 2025-26 funding update as of 31 October 2025. 2025. Available from:

https://reliefweb.int/report/bangladesh/bangladesh-rohingya-humanitarian-crisis-joint-response-plan-2025-26-funding-update-31-october-2025.

36. United Nations Children's Fund (UNICEF). Humanitarian Action for Children 2026 - Bangladesh. 2026. Available from: http://reliefweb.int/report/bangladesh/humanitarian-action-children-2026-bangladesh.

Supporting information

S1 Document. STROBE checklist for cross-sectional studies.

Dear Editor and Reviewer,

Thank you for reviewing our manuscript, on behalf of my co-authors - we sincerely appreciate the time you have spent to review our research manuscript. We have carefully reviewed each item raised during the peer review process and revised the manuscript accordingly.

Please see the line-by-line responses and revisions below:

Reviewer Comments	Author's Response
Reviewer #1: GENERAL	
- The data was collected in 2023, but things have changed dramatically for Rohingya families in Bangladesh. It would be useful to contextualize the results in light of the current difficulties in 2026, when the paper will be published.	• Yes, we acknowledge the gap between the date of data collection (late 2023) and the timing of our research manuscript under review. Anticipating such challenges, a pre-print of our research was provided to local authorities in advance to best inform programming efforts prior to peer-review. As such, the purpose of publication is more aligned with disseminating our research findings and discussing how these learnings should shape the international conceptualisation of education-in-emergencies similar to the Rohingya Refugee crisis rather than directly informing the current response.<br>• We have included an additional seven citations containing more recent evidence from the literature that has emerged since our first submission and key peer-review resources that better align our findings with the operational transition from acute crisis to enduring response and contemporary difficulties that confront local authorities in 2026. This includes:<br>○ The Inter-Sector Coordination Group's updated response plan published on May 20th 2026.<br>○ Recent World Health guidelines regarding have been cited to reinforce how the developmental period between childhood and adolescence is particularly crucial for teenage girls, significantly influencing their physical, psychosocial, and emotional well-being that persists into adulthood.<br>○ Recent World Health report regarding progress on the global action plan for promoting the health of refugees and migrants has been cited to link displacement and statelessness with their detrimental health impacts.<br>○ Four peer-review articles that discuss employment rights, sociocultural aspects, sexual and reproductive health among teenage girls, and their psychosocial impacts.
- The paper does not address quality of education in any substantive way. The severity classification system it uses captures access, learning conditions, protection factors, and aggravating circumstances. "Learning conditions" is treated narrowly, focused on whether the environment is physically safe and whether a child is enrolled, not on pedagogical quality, curriculum depth, or how the education compares to what families experienced before displacement.	• The severity classification system used (Interoperable Sectoral Severity Scale, AND Multi-Sectoral Needs Index) have been purposefully designed by UNICEF and Save the Children for data collected during Multi-Sectoral Needs Assessments such as this. Deviating from the purpose-built severity scale may otherwise compromise the construct validity of the ordinal outcome and therefore undermine our findings rather than enhance them.<br>• This classification scheme is predominantly protection based but also encompasses key elements of pedagogical quality ("lack of qualified teaching staff), curriculum depth ("age-wise curriculum unavailable", "lack of teaching and learning material"), and how the education compared to what families experienced before displacement ("curriculum not adapted"). These response items were mostly consolidated into the inadequate learning conditions variable for simplicity due to very rare occurrence rather than omitted from the assessment. The methods section has been revised to explicitly articulate the response items used to classify inadequate learning conditions to mitigate such concerns.

- The Bangladeshi government's restriction on formal education beyond level 3 for Rohingya children is a fundamental structural constraint that is not adequately acknowledged. Children classified as "stressed" — the best possible outcome — are still only accessing non-formal, NGO-run education with no pathway to formal credentialing. The sharp drop-off in enrollment after age 11 is a central finding of the paper, and the authors attribute it to gendered barriers, household survival pressures, and protection risks. Those are real factors. However, the government's grade 3 ceiling likely also contributes to families' decisions to disengage from the non-formal system as children get older, since there is no formal progression or credential to work toward. The policy context is especially relevant to the discussion of teenage boys and the "education is not a priority" reason code. Families making decisions under constraint (with no formal educational pathway available) may rationally conclude that continued enrollment in a dead-end non-formal system is not worth the opportunity cost for older children.	• We are aware of this policy, and this is what we intended by "systemic challenges" when discussing the main results in line 320-323 in the original manuscript. We have revised this sentence to explicitly note this restrictive enrolment policy that may have provoked the increased educational severity scores from age 11 onwards.<br>• I am reluctant to make this a central interpretation however as the empirical analysis over increasing age illustrated linear declines from age 11 onwards rather than an immediate drop-off that the ceiling effect from this restrictive policy environment would likely impose.<br>• Additional disaggregation by attributable reason such as "Too Old" and "Unable to Register or Enrol Child in School" did not demonstrate a sudden impact at age 11. Strong effect heterogeneity was also observed between teenage boys and teenage girls despite both being exposed to the same restriction.<br>• This policy consideration was another reason behind the selection of age-groupings to prevent contamination in quantitative analysis by distinguishing primary school aged and teenage children. The logistic regression results showed that teenage girls were associated with dramatically increased odds of higher educational severity scores relative to teenage boys. While we cannot be certain of why no such ceiling effect was observed from the proposed policy, we have empirical evidence of gender-specific factors that likely emerge around the same time in adolescence at age 11 and would explain the linear trends observed as they intensify with increasing age.<br>• As such, we have contextualised the decline observed at age 11 onwards through a theme of gendered interactions with the education sector rather than evidence of the restrictive policy's impact. Asserting otherwise may be perceived as contradictory to our findings and a prejudiced interpretation of our results rather than accurate contextualisation.<br>• Regarding the disengagement from the education system due to no formal education pathway, a similar dead-end argument could be made related to limited economic opportunities to utilise formal educational attainment. We have purposefully focused on the constraints that position households and adolescents to make such decisions rather than the decisions themselves. This is commensurate with our findings related to teenage girls and associations with household vulnerability factors. Conversely, we do not have the data necessary to elucidate individual perceptions of non-formal education system in such detail and at a representative response level in the same way we can relate household vulnerabilities to deprioritisation with empirical justification based on descriptive trends and quantitative multivariate analysis.
INTRODUCTION & LITERATURE REVIEW	
- The literature draws heavily on grey literature, which is OK. But it would be useful to also include peer-reviewed literature on education in emergencies, particularly on gendered barriers among Rohingya or comparable populations.	• Four citations of peer-review articles have been added that discuss employment rights, sociocultural aspects, gendered barriers to education, sexual and reproductive health among teenage girls, and their psychosocial impacts where appropriate<br>• Two World Health publications have been cited in the introduction to further evidence the epidemiological links between:<br>o Gender and widening health disparities in protracted conflict/displacement settings.<br>o Protective effects of education and early marriage/pregnancy among adolescent girls in low to middle income countries.
- The claim that the transition to adolescence "intensifies gendered effects" is asserted rather than evidenced at this point.	• We have softened our language to be more hypothetical rather than assertive and integrated additional supportive evidence from the literature.<br>• We have revised this point rather than removed it as it provides the conceptual foundations for our hypothesis and analytical rationale to disaggregate the results by gender that are key findings.
METHODOLOGY	
- The reliance on proxy respondents is a significant methodological limitation that is acknowledged but	• We have revised the first sentence in the method's variables section regarding the primary outcome to explicitly state that educational

somewhat underplayed in the methods section itself. It should be flagged earlier and more prominently, not just in the limitations.	severity was assigned based on an adult proxy respondent who answered on behalf of each education age child within the household.
- The rationale for age-group cutoffs (3–4, 5–11, 12–18) is partially justified by data trends, but the clinical/developmental logic is not spelled out. There is such variation, and there seem to be high rates of developmental issues among children (due to pregnancy-related complications or malnutrition), which should be taken into account.	• We have revised our method to reinforced the rationale for the age-groupings to prevent contamination caused by grouping heterogeneous age cohorts.<br>• We have added "developmental issues" to the limitations section as an example of unmeasured confounding in the discussion.<br>• "Disability" and "illness" were possible response items used to assign individual educational severity scores. These were infrequently reported.<br>• This also highlights the intersections between sectors where early marriage and teenage pregnancies interact with fragile antenatal, postnatal, maternal and neonatal health services to reinforce such issues.
- Income quartiles as a proxy for financial vulnerability is reasonable, but the paper does not report what those quartile thresholds actually are in dollar terms, which limits interpretability.	• We have updated the corresponding row in the main results table to include monetary values in USD rather than local currency units (Bangladeshi Taka) to align with how monetary values are reported by local humanitarian authorities using the same conversion rate from October 2023 World Food Programme reports among Rohingya refugees.
FINDINGS	
- The reported reasons for non-attendance (marriage/pregnancy, household labor, protection risks) give the numbers real-world grounding.	• Thank you
- Figures 1A, 1B, 2A, and 2B are well-constructed and carry a lot of the analytical weight effectively. The results section describes the figures at length in prose, which becomes repetitive. Some of that description could be condensed in favor of sharper interpretive framing.	• We have revised the manuscript text to focus the trend and let the illustration evidence the specifics rather than explicitly articulate them.
DISCUSSION AND CONCLUSION	
- The connection between household vulnerability factors (female-headed, elderly-headed households) and educational deprioritization under survival pressure is insightful and well-argued.	• Thank you
- The link to negative livelihood coping strategies is a useful framing.	• Thank you
- The discussion occasionally blurs the line between what the data showed and what the authors are interpreting. For example, the claim about "exogenous structural factors and systemic challenges" as an explanation for why individual-level associations persisted after adjustment is plausible but speculative. The paper would benefit from more explicit hedging in those moments.	• We have revised our language in the discussion section to be more suggestive rather than explicit claims.<br>• Our interpretation regarding exogenous structural factors and systemic challenges such as the aforementioned policy restriction serves to articulate a possible mechanism why such distinctive age-gender trends were observed, why household vulnerability factors were attenuated after adjustment for the child's individual age-group gender status, and the large effect size attributed to the child's age-group gender characteristics relative to the odds ratio's associated with household vulnerability factors.
- The reference to COVID-19 school closure effects feels somewhat grafted on; its relevance to 2023 data is not clearly established. It is my understanding that COVID-19 didn't impact the camps in the ways it was anticipated, but it would be useful to describe why that is important for the analysis.	• This has been removed to better focus on present challenges rather than historical issues.
LIMITATIONS	
- The limitations section is structured as a single dense paragraph. Separating the design limitations from the measurement limitations would improve readability. –	• Good point, we have separated these items for readability in the limitations section.
- The authors note that sensitive barriers "may be under-reported" but do not discuss the likely direction or magnitude of that bias on the main findings.	• We have revised this point to assert the potentially direction of lower odds ratios among girls that are more likely to experience both sensitivities and stigmatisation.
IMPLICATIONS & CONCLUSIONS	
- The connection to current humanitarian programming (school feeding, reuptake programs, female-only classrooms) is concrete and practically useful.	• Thank you
- "Protected crisis" or protracted crisis?	• Thank you, this was an error and has been corrected within the manuscript.

- The call for future longitudinal and qualitative research is appropriate but generic; the paper would be stronger if it specified what questions that research should answer and for whom.	• We have linked the need for longitudinal research with the limitations of causal inferences and prior comments in the discussion of main results regarding the arrangement of variables such as household vulnerability factors and individual age-gender characteristics that may be acting in causal pathways.<br>• We have also linked the need for qualitative research to mitigate against the sensitive protection-related barriers mentioned in the limitations section.

Regards,

Harry J Wilson (B.BMed.Sci, MPH, MIDI, MGH)

The Burnet Institute for Public Health and Medical Research

Monash University School of Public Health and Preventative Medicine
