# Supplementary material for: Prevalence of educational needs and factors associated with educational severity among Rohingya refugee children in Cox’s Bazar, Bangladesh: A representative cross-sectional household survey
Source: PLOS Glob Public Health. 2026 Aug 31;6(8):e0007223. doi: 10.1371/journal.pgph.0007223 (PMC13528982; doi:10.1371/journal.pgph.0007223)
Supplement: Supplementary file 3 [file pgph.0007223.s004.docx]

**Dear Editor and Reviewer,**

Thank you for reviewing our manuscript, on behalf of my co-authors - we sincerely appreciate the time you have spent to review our research manuscript. We have carefully reviewed each item raised during the peer review process and revised the manuscript accordingly.

Please see the line-by-line responses and revisions below:

| **Reviewer Comments** | **Author’s Response** |
| --- | --- |
| Reviewer #1: GENERAL |  |
| - The data was collected in 2023, but things have changed dramatically for Rohingya families in Bangladesh. It would be useful to contextualize the results in light of the current difficulties in 2026, when the paper will be published. | - Yes, we acknowledge the gap between the date of data collection (late 2023) and the timing of our research manuscript under review. Anticipating such challenges, a pre-print of our research was provided to local authorities in advance to best inform programming efforts prior to peer-review. As such, the purpose of publication is more aligned with disseminating our research findings and discussing how these learnings should shape the international conceptualisation of education-in-emergencies similar to the Rohingya Refugee crisis rather than directly informing the current response. - We have included an additional seven citations containing more recent evidence from the literature that has emerged since our first submission and key peer-review resources that better align our findings with the operational transition from acute crisis to enduring response and contemporary difficulties that confront local authorities in 2026. This includes:   - The Inter-Sector Coordination Group’s updated response plan published on May 20^th^ 2026.   - Recent World Health guidelines regarding have been cited to reinforce how the developmental period between childhood and adolescence is particularly crucial for teenage girls, significantly influencing their physical, psychosocial, and emotional well-being that persists into adulthood.   - Recent World Health report regarding progress on the global action plan for promoting the health of refugees and migrants has been cited to link displacement and statelessness with their detrimental health impacts.   - Four peer-review articles that discuss employment rights, sociocultural aspects, sexual and reproductive health among teenage girls, and their psychosocial impacts. |
| - The paper does not address quality of education in any substantive way. The severity classification system it uses captures access, learning conditions, protection factors, and aggravating circumstances. “Learning conditions" is treated narrowly, focused on whether the environment is physically safe and whether a child is enrolled, not on pedagogical quality, curriculum depth, or how the education compares to what families experienced before displacement. | - The severity classification system used (Interoperable Sectoral Severity Scale, AND Multi-Sectoral Needs Index) have been purposefully designed by UNICEF and Save the Children for data collected during Multi-Sectoral Needs Assessments such as this. Deviating from the purpose-built severity scale may otherwise compromise the construct validity of the ordinal outcome and therefore undermine our findings rather than enhance them. - This classification scheme is predominantly protection based but also encompasses key elements of pedagogical quality (“lack of qualified teaching staff), curriculum depth (“age-wise curriculum unavailable”, “lack of teaching and learning material”), and how the education compared to what families experienced before displacement (“curriculum not adapted”). These response items were mostly consolidated into the inadequate learning conditions variable for simplicity due to very rare occurrence rather than omitted from the assessment. The methods section has been revised to explicitly articulate the response items used to classify inadequate learning conditions to mitigate such concerns. |
| - The Bangladeshi government's restriction on formal education beyond level 3 for Rohingya children is a fundamental structural constraint that is not adequately acknowledged. Children classified as "stressed" — the best possible outcome — are still only accessing non-formal, NGO-run education with no pathway to formal credentialing. The sharp drop-off in enrollment after age 11 is a central finding of the paper, and the authors attribute it to gendered barriers, household survival pressures, and protection risks. Those are real factors. However, the government's grade 3 ceiling likely also contributes to families' decisions to disengage from the non-formal system as children get older, since there is no formal progression or credential to work toward. The policy context is especially relevant to the discussion of teenage boys and the "education is not a priority" reason code. Families making decisions under constraint (with no formal educational pathway available) may rationally conclude that continued enrollment in a dead-end non-formal system is not worth the opportunity cost for older children. | - We are aware of this policy, and this is what we intended by “systemic challenges” when discussing the main results in line 320-323 in the original manuscript. We have revised this sentence to explicitly note this restrictive enrolment policy that may have provoked the increased educational severity scores from age 11 onwards. - I am reluctant to make this a central interpretation however as the empirical analysis over increasing age illustrated linear declines from age 11 onwards rather than an immediate drop-off that the ceiling effect from this restrictive policy environment would likely impose. - Additional disaggregation by attributable reason such as “Too Old” and “Unable to Register or Enrol Child in School” did not demonstrate a sudden impact at age 11. Strong effect heterogeneity was also observed between teenage boys and teenage girls despite both being exposed to the same restriction. - This policy consideration was another reason behind the selection of age-groupings to prevent contamination in quantitative analysis by distinguishing primary school aged and teenage children. The logistic regression results showed that teenage girls were associated with dramatically increased odds of higher educational severity scores relative to teenage boys. While we cannot be certain of why no such ceiling effect was observed from the proposed policy, we have empirical evidence of gender-specific factors that likely emerge around the same time in adolescence at age 11 and would explain the linear trends observed as they intensify with increasing age. - As such, we have contextualised the decline observed at age 11 onwards through a theme of gendered interactions with the education sector rather than evidence of the restrictive policy’s impact. Asserting otherwise may be perceived as contradictory to our findings and a prejudiced interpretation of our results rather than accurate contextualisation. - Regarding the disengagement from the education system due to no formal education pathway, a similar dead-end argument could be made related to limited economic opportunities to utilise formal educational attainment. We have purposefully focused on the constraints that position households and adolescents to make such decisions rather than the decisions themselves. This is commensurate with our findings related to teenage girls and associations with household vulnerability factors. Conversely, we do not have the data necessary to elucidate individual perceptions of non-formal education system in such detail and at a representative response level in the same way we can relate household vulnerabilities to deprioritisation with empirical justification based on descriptive trends and quantitative multivariate analysis. |
|  |  |
| INTRODUCTION & LITERATURE REVIEW |  |
| - The literature draws heavily on grey literature, which is OK. But it would be useful to also include peer-reviewed literature on education in emergencies, particularly on gendered barriers among Rohingya or comparable populations. | - Four citations of peer-review articles have been added that discuss employment rights, sociocultural aspects, gendered barriers to education, sexual and reproductive health among teenage girls, and their psychosocial impacts where appropriate - Two World Health publications have been cited in the introduction to further evidence the epidemiological links between:   - Gender and widening health disparities in protracted conflict/displacement settings.   - Protective effects of education and early marriage/pregnancy among adolescent girls in low to middle income countries. |
| - The claim that the transition to adolescence "intensifies gendered effects" is asserted rather than evidenced at this point. | - We have softened our language to be more hypothetical rather than assertive and integrated additional supportive evidence from the literature. - We have revised this point rather than removed it as it provides the conceptual foundations for our hypothesis and analytical rationale to disaggregate the results by gender that are key findings. |
|  |  |
| METHODOLOGY |  |
| - The reliance on proxy respondents is a significant methodological limitation that is acknowledged but somewhat underplayed in the methods section itself. It should be flagged earlier and more prominently, not just in the limitations. | - We have revised the first sentence in the method’s variables section regarding the primary outcome to explicitly state that educational severity was assigned based on an adult proxy respondent who answered on behalf of each education age child within the household. |
| - The rationale for age-group cutoffs (3–4, 5–11, 12–18) is partially justified by data trends, but the clinical/developmental logic is not spelled out. There is such variation, and there seem to be high rates of developmental issues among children (due to pregnancy-related complications or malnutrition), which should be taken into account. | - We have revised our method to reinforced the rationale for the age-groupings to prevent contamination caused by grouping heterogeneous age cohorts. - We have added “developmental issues” to the limitations section as an example of unmeasured confounding in the discussion. - “Disability” and “illness” were possible response items used to assign individual educational severity scores. These were infrequently reported. - This also highlights the intersections between sectors where early marriage and teenage pregnancies interact with fragile antenatal, postnatal, maternal and neonatal health services to reinforce such issues. |
| - Income quartiles as a proxy for financial vulnerability is reasonable, but the paper does not report what those quartile thresholds actually are in dollar terms, which limits interpretability. | - We have updated the corresponding row in the main results table to include monetary values in USD rather than local currency units (Bangladeshi Taka) to align with how monetary values are reported by local humanitarian authorities using the same conversion rate from October 2023 World Food Programme reports among Rohingya refugees. |
|  |  |
| FINDINGS |  |
| - The reported reasons for non-attendance (marriage/pregnancy, household labor, protection risks) give the numbers real-world grounding. | - Thank you |
| - Figures 1A, 1B, 2A, and 2B are well-constructed and carry a lot of the analytical weight effectively. The results section describes the figures at length in prose, which becomes repetitive. Some of that description could be condensed in favor of sharper interpretive framing. | - We have revised the manuscript text to focus the trend and let the illustration evidence the specifics rather than explicitly articulate them. |
|  |  |
| DISCUSSION AND CONCLUSION |  |
| - The connection between household vulnerability factors (female-headed, elderly-headed households) and educational deprioritization under survival pressure is insightful and well-argued. | - Thank you |
| - The link to negative livelihood coping strategies is a useful framing. | - Thank you |
| - The discussion occasionally blurs the line between what the data showed and what the authors are interpreting. For example, the claim about "exogenous structural factors and systemic challenges" as an explanation for why individual-level associations persisted after adjustment is plausible but speculative. The paper would benefit from more explicit hedging in those moments. | - We have revised our language in the discussion section to be more suggestive rather than explicit claims. - Our interpretation regarding exogenous structural factors and systemic challenges such as the aforementioned policy restriction serves to articulate a possible mechanism why such distinctive age-gender trends were observed, why household vulnerability factors were attenuated after adjustment for the child’s individual age-group gender status, and the large effect size attributed to the child’s age-group gender characteristics relative to the odds ratio’s associated with household vulnerability factors. |
| - The reference to COVID-19 school closure effects feels somewhat grafted on; its relevance to 2023 data is not clearly established. It is my understanding that COVID-19 didn’t impact the camps in the ways it was anticipated, but it would be useful to describe why that is important for the analysis. | - This has been removed to better focus on present challenges rather than historical issues. |
|  |  |
| LIMITATIONS |  |
| - The limitations section is structured as a single dense paragraph. Separating the design limitations from the measurement limitations would improve readability. – | - Good point, we have separated these items for readability in the limitations section. |
| - The authors note that sensitive barriers "may be under-reported" but do not discuss the likely direction or magnitude of that bias on the main findings. | - We have revised this point to assert the potentially direction of lower odds ratios among girls that are more likely to experience both sensitivities and stigmatisation. |
|  |  |
| IMPLICATIONS & CONCLUSIONS |  |
| - The connection to current humanitarian programming (school feeding, reuptake programs, female-only classrooms) is concrete and practically useful. | - Thank you |
| - “Protected crisis” or protracted crisis? | - Thank you, this was an error and has been corrected within the manuscript. |
| - The call for future longitudinal and qualitative research is appropriate but generic; the paper would be stronger if it specified what questions that research should answer and for whom. | - We have linked the need for longitudinal research with the limitations of causal inferences and prior comments in the discussion of main results regarding the arrangement of variables such as household vulnerability factors and individual age-gender characteristics that may be acting in causal pathways. - We have also linked the need for qualitative research to mitigate against the sensitive protection-related barriers mentioned in the limitations section. |

**Regards,**

**Harry J Wilson (B.BMed.Sci, MPH, MIDI, MGH)**

The Burnet Institute for Public Health and Medical Research

Monash University School of Public Health and Preventative Medicine
