# Supplementary material for: Prevalence of educational needs and factors associated with educational severity among Rohingya refugee children in Cox’s Bazar, Bangladesh: A representative cross-sectional household survey
Source: PLOS Glob Public Health. 2026 Aug 31;6(8):e0007223. doi: 10.1371/journal.pgph.0007223 (PMC13528982; doi:10.1371/journal.pgph.0007223)
Supplement: Supplementary file 4 [file pgph.0007223.s005.docx]

**Dear Editor and Reviewers,**

Thank you for reviewing our revised manuscript - we sincerely appreciate the time you have spent to strengthen our research throughout the publication process. We have carefully reviewed the outstanding items raised during the second round of peer review and have revised the manuscript accordingly.

Please see the line-by-line responses and revisions below:

| **Reviewer Comments** | **Author’s Response** |
| --- | --- |
| Reviewer #1: Point 1 |  |
| The Bangladesh government's restriction on formal education beyond grade 3 remains unacknowledged as a named policy. The revision adds the phrase "restrictive enrolment policies experienced by teenage children" to the Discussion. However, it is too vague to constitute adequate treatment. The specific policy, its implications for the non-formal education ceiling, and its likely role in shaping household decision-making about older children's schooling are not discussed. This is particularly important for interpreting the "education is not a priority" reason code among teenage boys, which the paper attributes to household survival pressure without considering that families may be responding rationally to a system with no formal pathway or credential to work toward. | - We have revised this point in our discussion to specifically include the reviewer’s comments. We have explicitly listed the “government’s restrictive enrolment policy beyond grade 3 that may provoke the increasing trend in educational severity scores observed from age 11 onwards”. - We have further elaborated on the implications of the “non-formal education ceiling” in our discussion section as requested. - We have integrated the reviewer’s specific comment regarding education is not a priority among teenage boys and rational behaviour from the household that are disengaged from the non-formal education system. - Please note that the purpose behind why we have suggested that deprioritisation may be associated with household survival pressures served to contextualise why household vulnerability factors were associated with higher odds of increasing educational severity during logistic regression analysis by postulating a potential mechanism through which these variables may act. |
| Reviewer #1: Point 2 |  |
| The age-group cutoff rationale has not been substantively addressed. The revision adds one sentence noting that the 5-11 and 12-18 cutpoint was chosen "to mitigate contamination of age-groups from effect heterogeneity observed during linear trend analysis." This is a statistical justification, not a developmental one. The concern about high rates of developmental variation among this population due to malnutrition and pregnancy-related complications is mentioned only fleetingly in the limitations as "complex developmental issues that may modulate individual abilities to engage with the education sector." This is a substantive methodological issue that should be addressed. | - We have added additional methods defence to justify the selected age-group cutpoints to encompass statistical, operational, sector-specific (curriculum) and developmental rationale as follows:  1. Statistically: prevent age-group specific contamination of heterogeneity observed during linear trend analysis 2. Operationally: alignment with age-group education sector reporting by the local humanitarian authority in Cox’s Bazar (The Intersectoral Coordination Group (ISCG)). See ISCG camp-level findings (manuscript reference 30). Note that these results are derived from the same deidentified participant dataset underlying our own results.    a. The report provides education sector estimates and camp-level findings disaggregated by gender (boys, girls, children) and age-group (5-11, 12-18, 5-18), in accordance with our results provided in figure 1A and 1B.    b. See highlights summarising results for all camps (page 4 - ISCG camp-level findings; manuscript reference 30)      c. See age-group classifications highlighted from camp-level findings (page 8 - ISCG camp-level findings; manuscript reference 30)      d. A summary of humanitarian indicators by camp is provided on page 106-108. Within the education sector, the two indicators reported are:    i. % of children (5-18 y.o.) reportedly enrolled and attending camps' education facilities    ii. % of children aged 3-4 y.o. who attended any early childhood education programme   See highlighted indicators (page 106 - ISCG camp-level findings; manuscript reference 30)     3. Education Sector Curriculum:    a. Age 3-4 early learning    b. Age 5-11, primary school (kindergarten to grade 5)    c. Age 12-18, middle and secondary school (grade 6-12) 4. Developmental: Using gender-specific reason codes as markers of development among school-age children:    a. age 12 was the youngest age reported for childhood marriage/early pregnancy as seen in Figure 2B. This suggests an age-specific floor effect where exposure begins at age 12.    b. Inadequate Male-Female Separation rapidly diverged at age 12 for girls and further increased over the age range. This also suggests an age-specific exposure that starts at age 12.  - We believe that our selection provides the best balance between these competing factors of statistical contamination, operational alignment, education sector curriculum, and developmental criteria as inferred from the onset of gender-specific response items. - We have further elaborated on the issue regarding “high rates of developmental variation among this population” as requested.   - We have specifically included their relationship with “malnutrition and pregnancy-related complications” as suggested. We have added two additional citations to substantiate this claim: Tsichlis et al. Malnutrition trends in displaced Rohingya children from 2017 to 2024: a comprehensive 7-year repeated cross-sectional analysis. BMJ Glob Health. 2026   Simon et al. Predictors of antenatal care utilization among the Rohingya population in the refugee camps of Cox's Bazar, Bangladesh. Confl Health. 2025   - We have further discussed the generalisability of our research to external humanitarian scenarios with different developmental characteristics. - We have also suggested further qualitative research to investigate how developmental complexities interact with the education system to better inform prospective programming efforts. |
| Reviewer #2: Point 1 |  |
| I did not see any mention of education in the keywords, but I believe it is important to include it. | We agree and have added “Education” as a keyword to both the manuscript (end of abstract section) and submission documentation for indexing. |

**Regards,**

**Harry J Wilson (B.BMed.Sci, MPH, MIDI, MGH)**

The Burnet Institute for Public Health and Medical Research

Monash University School of Public Health and Preventative Medicine
