# Supplementary material for: Prevalence of educational needs and factors associated with educational severity among Rohingya refugee children in Cox’s Bazar, Bangladesh: A representative cross-sectional household survey
Source: PLOS Glob Public Health. 2026 Aug 31;6(8):e0007223. doi: 10.1371/journal.pgph.0007223 (PMC13528982; doi:10.1371/journal.pgph.0007223)
Supplement: Peer review history

# Peer review: Decision Letter 0

**DOI:** 10.1371/journal.pgph.0007223.r001

**Authors:** Katrien Janin

28 May 2026

PGPH-D-26-00678

Prevalence of educational needs and factors associated with educational severity among Rohingya refugee children in Cox's Bazar, Bangladesh: a representative cross-sectional household survey

PLOS Global Public Health

Dear Dr. Wilson,

Thank you for submitting your manuscript to PLOS Global Public Health. After careful consideration, we feel that it has merit but does not fully meet PLOS Global Public Health’s publication criteria as it currently stands. Therefore, we invite you to submit a revised version of the manuscript that addresses the points raised during the review process.

Please note that we have only been able to secure a single reviewer to assess your manuscript. We are issuing a decision on your manuscript at this point to prevent further delays in the evaluation of your manuscript. Please be aware that the editor who handles your revised manuscript might find it necessary to invite additional reviewers to assess this work once the revised manuscript is submitted. However, we will aim to proceed on the basis of this single review if possible.

The reviewer has provided a detailed assessment of your manuscript below. Please take these points into careful consideration when preparing your revised manuscript and your point‑by‑point response.

Please submit your revised manuscript by Jul 27 2026 11:59PM. If you will need more time than this to complete your revisions, please reply to this message or contact the journal office at globalpubhealth@plos.org. When you're ready to submit your revision, log on to https://www.editorialmanager.com/pgph/ and select the 'Submissions Needing Revision' folder to locate your manuscript file.

Please include the following items when submitting your revised manuscript:

- A letter that responds to each point raised by the editor and reviewer(s). You should upload this letter as a separate file labeled 'Response to Reviewers'.
- A marked-up copy of your manuscript that highlights changes made to the original version. You should upload this as a separate file labeled 'Revised Manuscript with Track Changes'.
- An unmarked version of your revised paper without tracked changes. You should upload this as a separate file labeled 'Manuscript'.

Guidelines for resubmitting your figure files are available below the reviewer comments at the end of this letter.

As the corresponding author, your ORCID iD is verified in the submission system and will appear in the published article. PLOS supports the use of ORCID, and we encourage all coauthors to register for an ORCID iD and use it as well. Please encourage your coauthors to verify their ORCID iD within the submission system before final acceptance, as unverified ORCID iDs will not appear in the published article. *Only*  the individual author can complete the verification step; PLOS staff *cannot*  verify ORCID iDs on behalf of authors.

We look forward to receiving your revised manuscript.

Kind regards,

Katrien G. Janin, PhD

Staff Editor

PLOS Global Public Health

Journal Requirements:

1. Please ensure that your Ethics Statement is available in its entirety at the beginning of your Methods section, under a subheading 'Ethics Statement'.

2. In the online submission form, you indicated that "The de-identified participant-level dataset underlying the study results may be available upon reasonable request to UNHCR at https://microdata.unhcr.org/index.php/catalog/1128 subject to approval based on their data sharing policies, ethical requirements, and data use agreements.".

All PLOS journals now require all data underlying the findings described in their manuscript to be freely available to other researchers, either

1. In a public repository,

2. Within the manuscript itself, or

3. Uploaded as supplementary information.

This policy applies to all data except where public deposition would breach compliance with the protocol approved by your research ethics board. If your data cannot be made publicly available for ethical or legal reasons (e.g., public availability would compromise patient privacy), please explain your reasons by return email and your exemption request will be escalated to the editor for approval. Your exemption request will be handled independently and will not hold up the peer review process, but will need to be resolved should your manuscript be accepted for publication. One of the Editorial team will then be in touch if there are any issues.

If the reviewer comments include a recommendation to cite specific previously published works, please review and evaluate these publications to determine whether they are relevant and should be cited. There is no requirement to cite these works unless the editor has indicated otherwise.

Additional Editor Comments (if provided):

[Note: HTML markup is below. Please do not edit.]

Reviewers' comments:

Reviewer's Responses to Questions

**Comments to the Author**

1. Does this manuscript meet PLOS Global Public Health’s publication criteria?

Reviewer #1: Yes

**********

2. Has the statistical analysis been performed appropriately and rigorously?

Reviewer #1: I don't know

**********

3. Have the authors made all data underlying the findings in their manuscript fully available (please refer to the Data Availability Statement at the start of the manuscript PDF file)?

The PLOS Data policy

Reviewer #1: Yes

**********

4. Is the manuscript presented in an intelligible fashion and written in standard English?

Reviewer #1: Yes

**********

Reviewer #1: GENERAL

- The data was collected in 2023, but things have changed dramatically for Rohingya families in Bangladesh. It would be useful to contextualize the results in light of the current difficulties in 2026, when the paper will be published.

- The paper does not address quality of education in any substantive way. The severity classification system it uses captures access, learning conditions, protection factors, and aggravating circumstances. “Learning conditions" is treated narrowly, focused on whether the environment is physically safe and whether a child is enrolled, not on pedagogical quality, curriculum depth, or how the education compares to what families experienced before displacement.

- The Bangladeshi government's restriction on formal education beyond level 3 for Rohingya children is a fundamental structural constraint that is not adequately acknowledged. Children classified as "stressed" — the best possible outcome — are still only accessing non-formal, NGO-run education with no pathway to formal credentialing. The sharp drop-off in enrollment after age 11 is a central finding of the paper, and the authors attribute it to gendered barriers, household survival pressures, and protection risks. Those are real factors. However, the government's grade 3 ceiling likely also contributes to families' decisions to disengage from the non-formal system as children get older, since there is no formal progression or credential to work toward. The policy context is especially relevant to the discussion of teenage boys and the "education is not a priority" reason code. Families making decisions under constraint (with no formal educational pathway available) may rationally conclude that continued enrollment in a dead-end non-formal system is not worth the opportunity cost for older children.

INTRODUCTION & LITERATURE REVIEW

- The literature draws heavily on grey literature, which is OK. But it would be useful to also include peer-reviewed literature on education in emergencies, particularly on gendered barriers among Rohingya or comparable populations.

- The claim that the transition to adolescence "intensifies gendered effects" is asserted rather than evidenced at this point.

METHODOLOGY

- The reliance on proxy respondents is a significant methodological limitation that is acknowledged but somewhat underplayed in the methods section itself. It should be flagged earlier and more prominently, not just in the limitations.

- The rationale for age-group cutoffs (3–4, 5–11, 12–18) is partially justified by data trends, but the clinical/developmental logic is not spelled out. There is such variation, and there seem to be high rates of developmental issues among children (due to pregnancy-related complications or malnutrition), which should be taken into account.

- Income quartiles as a proxy for financial vulnerability is reasonable, but the paper does not report what those quartile thresholds actually are in dollar terms, which limits interpretability.

FINDINGS

- The reported reasons for non-attendance (marriage/pregnancy, household labor, protection risks) give the numbers real-world grounding.

- Figures 1A, 1B, 2A, and 2B are well-constructed and carry a lot of the analytical weight effectively. The results section describes the figures at length in prose, which becomes repetitive. Some of that description could be condensed in favor of sharper interpretive framing.

DISCUSSION AND CONCLUSION

- The connection between household vulnerability factors (female-headed, elderly-headed households) and educational deprioritization under survival pressure is insightful and well-argued.

- The link to negative livelihood coping strategies is a useful framing.

- The discussion occasionally blurs the line between what the data showed and what the authors are interpreting. For example, the claim about "exogenous structural factors and systemic challenges" as an explanation for why individual-level associations persisted after adjustment is plausible but speculative. The paper would benefit from more explicit hedging in those moments.

- The reference to COVID-19 school closure effects feels somewhat grafted on; its relevance to 2023 data is not clearly established. It is my understanding that COVID-19 didn’t impact the camps in the ways it was anticipated, but it would be useful to describe why that is important for the analysis.

LIMITATIONS

- The limitations section is structured as a single dense paragraph. Separating the design limitations from the measurement limitations would improve readability. –

- The authors note that sensitive barriers "may be under-reported" but do not discuss the likely direction or magnitude of that bias on the main findings.

IMPLICATIONS & CONCLUSIONS

- The connection to current humanitarian programming (school feeding, reuptake programs, female-only classrooms) is concrete and practically useful.

- “Protected crisis” or protracted crisis?

- The call for future longitudinal and qualitative research is appropriate but generic; the paper would be stronger if it specified what questions that research should answer and for whom.

**********

what does this mean?). If published, this will include your full peer review and any attached files.

**Do you want your identity to be public for this peer review?**  If you choose “no”, your identity will remain anonymous but your review may still be made public.

For information about this choice, including consent withdrawal, please see our Privacy Policy

Reviewer #1: **Yes:** Bree Akesson

**********

[NOTE: If reviewer comments were submitted as an attachment file, they will be attached to this email and accessible via the submission site. Please log into your account, locate the manuscript record, and check for the action link "View Attachments". If this link does not appear, there are no attachment files.]

# Author comment: Author response to Decision Letter 1

**DOI:** 10.1371/journal.pgph.0007223.r002

9 Jun 2026

Attachment Submitted filename: Response to Reviewers.docx

# Peer review: Decision Letter 1

**DOI:** 10.1371/journal.pgph.0007223.r003

**Authors:** M. A. Rifat

5 Aug 2026

PGPH-D-26-00678R1

Prevalence of educational needs and factors associated with educational severity among Rohingya refugee children in Cox's Bazar, Bangladesh: a representative cross-sectional household survey

PLOS Global Public Health

Dear Dr. Harry Wilson,

Thank you for submitting your manuscript to PLOS Global Public Health. Based on comments from reviewers, we feel that your manuscript requires a minor revision to fully meet PLOS Global Public Health’s publication criteria. Therefore, we invite you to submit a revised version of the manuscript that addresses the points raised during the review process.

Please submit your revised manuscript at your earliest convenient and no later than 31/08/2026. If you will need more time than this to complete your revisions, please reply to this message or contact the journal office at globalpubhealth@plos.org. When you're ready to submit your revision, log on to https://www.editorialmanager.com/pgph/ and select the 'Submissions Needing Revision' folder to locate your manuscript file.

Please include the following items when submitting your revised manuscript:

- A letter that responds to each point raised by the editor and reviewer(s). You should upload this letter as a separate file labeled 'Response to Reviewers'.
- A marked-up copy of your manuscript that highlights changes made to the original version. You should upload this as a separate file labeled 'Revised Manuscript with Track Changes'.
- An unmarked version of your revised paper without tracked changes. You should upload this as a separate file labeled 'Manuscript'.

Guidelines for resubmitting your figure files are available below the reviewer comments at the end of this letter.

As the corresponding author, your ORCID iD is verified in the submission system and will appear in the published article. PLOS supports the use of ORCID, and we encourage all coauthors to register for an ORCID iD and use it as well. Please encourage your coauthors to verify their ORCID iD within the submission system before final acceptance, as unverified ORCID iDs will not appear in the published article. *Only*  the individual author can complete the verification step; PLOS staff *cannot*  verify ORCID iDs on behalf of authors.

We look forward to receiving your revised manuscript.

Kind regards,

M. A. Rifat

Academic Editor

PLOS Global Public Health

Journal Requirements:

If the reviewer comments include a recommendation to cite specific previously published works, please review and evaluate these publications to determine whether they are relevant and should be cited. There is no requirement to cite these works unless the editor has indicated otherwise.

Please review your reference list to ensure that it is complete and correct. If you have cited papers that have been retracted, please include the rationale for doing so in the manuscript text, or remove these references and replace them with relevant current references. Any changes to the reference list should be mentioned in the rebuttal letter that accompanies your revised manuscript. If you need to cite a retracted article, indicate the article’s retracted status in the References list and also include a citation and full reference for the retraction notice.

Additional Editor Comments (if provided):

[Note: HTML markup is below. Please do not edit.]

Reviewers' comments:

Reviewer's Responses to Questions

**Comments to the Author**

Reviewer #1: (No Response)

Reviewer #2: All comments have been addressed

**********

publication criteria?

Reviewer #1: Yes

Reviewer #2: Yes

**********

3. Has the statistical analysis been performed appropriately and rigorously?

Reviewer #1: I don't know

Reviewer #2: Yes

**********

4. Have the authors made all data underlying the findings in their manuscript fully available (please refer to the Data Availability Statement at the start of the manuscript PDF file)?

The PLOS Data policy

Reviewer #1: Yes

Reviewer #2: Yes

**********

5. Is the manuscript presented in an intelligible fashion and written in standard English?

Reviewer #1: Yes

Reviewer #2: Yes

**********

Reviewer #1: The manuscript is much improved and it seems like the authors have addressed most of my comments. However, there reamin 2 areas that I would really like to see addressed before publication:

1. The Bangladesh government's restriction on formal education beyond grade 3 remains unacknowledged as a named policy. The revision adds the phrase "restrictive enrolment policies experienced by teenage children" to the Discussion. However, it is too vague to constitute adequate treatment. The specific policy, its implications for the non-formal education ceiling, and its likely role in shaping household decision-making about older children's schooling are not discussed. This is particularly important for interpreting the "education is not a priority" reason code among teenage boys, which the paper attributes to household survival pressure without considering that families may be responding rationally to a system with no formal pathway or credential to work toward.

2. The age-group cutoff rationale has not been substantively addressed. The revision adds one sentence noting that the 5-11 and 12-18 cutpoint was chosen "to mitigate contamination of age-groups from effect heterogeneity observed during linear trend analysis." This is a statistical justification, not a developmental one. The concern about high rates of developmental variation among this population due to malnutrition and pregnancy-related complications is mentioned only fleetingly in the limitations as "complex developmental issues that may modulate individual abilities to engage with the education sector." This is a substantive methodological issue that should be addressed.

Reviewer #2: I did not see any mention of education in the keywords, but I believe it is important to include it.

**********

what does this mean?). If published, this will include your full peer review and any attached files.

**Do you want your identity to be public for this peer review?**  If you choose “no”, your identity will remain anonymous but your review may still be made public.

For information about this choice, including consent withdrawal, please see our Privacy Policy

Reviewer #1: **Yes:** Bree Akesson

Reviewer #2: No

**********

[NOTE: If reviewer comments were submitted as an attachment file, they will be attached to this email and accessible via the submission site. Please log into your account, locate the manuscript record, and check for the action link "View Attachments". If this link does not appear, there are no attachment files.]

Attachment Submitted filename: PGPH-D-26-00678_R1 complete.pdf

# Author comment: Author response to Decision Letter 2

**DOI:** 10.1371/journal.pgph.0007223.r004

10 Aug 2026

Attachment Submitted filename: Response_to_Reviewers_auresp_2.docx

# Editor report: Decision Letter 2

**DOI:** 10.1371/journal.pgph.0007223.r005

**Authors:** M. A. Rifat

14 Aug 2026

Prevalence of educational needs and factors associated with educational severity among Rohingya refugee children in Cox's Bazar, Bangladesh: a representative cross-sectional household survey

PGPH-D-26-00678R2

Dear Dr. Harry J Wilson,

We are pleased to inform you that your manuscript 'Prevalence of educational needs and factors associated with educational severity among Rohingya refugee children in Cox's Bazar, Bangladesh: a representative cross-sectional household survey' has been provisionally accepted for publication in PLOS Global Public Health.

Before your manuscript can be formally accepted you will need to complete some formatting changes, which you will receive in a follow up email. A member of our team will be in touch with a set of requests.

Please note that your manuscript will not be scheduled for publication until you have made the required changes, so a swift response is appreciated.

IMPORTANT: The editorial review process is now complete. PLOS will only permit corrections to spelling, formatting or significant scientific errors from this point onwards. Requests for major changes, or any which affect the scientific understanding of your work, will cause delays to the publication date of your manuscript.

If your institution or institutions have a press office, please notify them about your upcoming paper to help maximize its impact. If they'll be preparing press materials, please inform our press team as soon as possible -- no later than 48 hours after receiving the formal acceptance. Your manuscript will remain under strict press embargo until 2 pm Eastern Time on the date of publication. For more information, please contact globalpubhealth@plos.org.

Thank you again for supporting Open Access publishing; we are looking forward to publishing your work in PLOS Global Public Health.

Best regards,

M. A. Rifat

Academic Editor

PLOS Global Public Health

***********************************************************

Reviewer Comments (if any, and for reference):
